# Human stem cells home to and repair laser-damaged trabecular meshwork in a mouse model

**DOI:** 10.1038/s42003-018-0227-z

**Published:** 2018-12-06

**Authors:** Hongmin Yun, Yiwen Wang, Yi Zhou, Ke Wang, Ming Sun, Donna B. Stolz, Xiaobo Xia, C. Ross Ethier, Yiqin Du

**Affiliations:** 1 Department of Ophthalmology, University of Pittsburgh, Pittsburgh, PA 15213 USA; 2 Department of Ophthalmology, Xiangya Hospital, Central South University, 410008 Changsha, Hunan China; 3 Department of Biomedical Engineering, Georgia Institute of Technology/Emory University, Atlanta, GA 30332 USA; 4 Department of Cell Biology, University of Pittsburgh, Pittsburgh, PA 15213 USA; 5 McGowan Institute for Regenerative Medicine, University of Pittsburgh, Pittsburgh, PA 15213 USA; 6 Department of Developmental Biology, University of Pittsburgh, Pittsburgh, PA 15213 USA

**Keywords:** Mesenchymal stem cells, Glaucoma

## Abstract

Glaucoma is the leading cause of irreversible vision loss, and reducing elevated intraocular pressure is currently the only effective clinical treatment. The trabecular meshwork is the main resistance site for aqueous outflow that maintains intraocular pressure. In this study, we transplanted human trabecular meshwork stem cells (TMSCs) intracamerally into mice that received laser photocoagulation over a 180° arc of the trabecular meshwork. TMSCs preferentially homed and integrated to the laser-damaged trabecular meshwork region and expressed differentiated cell markers at 2 and 4 weeks. Laser-induced inflammatory and fibrotic responses were prevented by TMSC transplantation with simultaneous ultrastructure and function restoration. Cell affinity and migration assays and elevated expression of CXCR4 and SDF1 in laser-treated mouse trabecular meshwork suggest that the CXCR4/SDF1 chemokine axis plays an important role in TMSC homing. Our results suggest that TMSCs may be a viable candidate for trabecular meshwork refunctionalization as a novel treatment for glaucoma.

## Abstract (web summary)

Hongmin Yun et al. show that implanted human stem cells can accurately home to and repair damaged trabecular meshwork tissue in the mouse eye via a chemokine axis defined by CXCR4 and SDF1. The study suggests that stem cells from the trabecular meshwork could be used to refunctionalize the outflow pathway as a treatment for glaucoma.

## Introduction

Glaucoma, a progressive optic neuropathy, is the leading cause of irreversible blindness worldwide^1^. The most common subtype of glaucoma is primary open-angle glaucoma, with about 45 million patients suffering from this condition worldwide^2^. Although nearly 40% of primary open-angle glaucoma patients may not have recorded elevated intraocular pressure (IOP)^2^, elevated IOP is still agreed to be a major risk factor; moreover, IOP lowering is currently the only effective clinical treatment for glaucoma. The primary cause of elevated IOP is impaired drainage of aqueous humor from the eye, i.e., a reduction in aqueous outflow facility. There are two pathways for aqueous outflow from the eye^3^. In the unconventional, or uveoscleral, outflow pathway, aqueous humor flows from the anterior chamber into the ciliary muscle before exiting the eye. In the conventional, or trabecular, outflow pathway, aqueous humor flows from the anterior chamber through the trabecular meshwork, Schlemm’s canal, and vessels connecting Schlemm’s canal to the episcleral veins. The trabecular meshwork consists of the uveal meshwork, corneoscleral meshwork, and juxtacanalicular connective tissue. It is believed that the juxtacanalicular region of the trabecular meshwork provides the main resistance to aqueous outflow^4^. Most anti-glaucoma treatments decrease IOP either by targeting the unconventional outflow pathway or by reducing the production of aqueous humor^2^. Recently some studies have focused on discovering new drugs that target the conventional outflow pathway, which is responsible for up to 90% of aqueous outflow^5^ and is the main cause of increased IOP in glaucoma.

It has been shown that reduced cellularity of the trabecular meshwork is associated with glaucoma and aging^6,7^ and that reduction of trabecular meshwork cellularity may be related to increased stiffness^8,9^ and trabecular beam fusion in aged^7^ and glaucomatous trabecular meshwork^10^. A myocilin (MYOC) mutant mouse glaucoma model^11,12^ demonstrating trabecular meshwork cell death and IOP elevation emphasizes the importance of trabecular meshwork cell function for normal aqueous outflow. Trabecular meshwork cells may also interact with Schlemm’s canal endothelial cells^13,14^, which also provide resistance to aqueous outflow.

Studies on human eyes that received laser trabeculoplasty^15^ showed that there was a population of trabecular meshwork cells that underwent increased cell division and migration to repopulate the damaged trabecular meshwork. This has motivated study into the use of stem cells to repopulate and refunctionalize the trabecular meshwork and hence reduce IOP in glaucoma patients. Stem cells are characterized by asymmetric cell division, self-renewal, and the ability to generate differentiated daughter cells. They are capable of multilineage differentiation and functional reconstruction of damaged tissues in vivo^16^. The ability of stem cells to maintain quiescence is critical for the long-term maintenance of a functional stem cell pool for regeneration, which represents one of the advantages of stem cells vs. differentiated cells in tissue regeneration.

It has been reported that there are tissue-specific stem cells in the trabecular meshwork^17–22^. Specifically, trabecular meshwork stem cells (TMSCs) are located in a niche under Schwalbe’s line and between the trabecular meshwork and the corneal endothelium^17–19,23^. Several groups, including ours, have successfully isolated and characterized human TMSCs^22,24–26^. These TMSCs have different gene marker expression profile compared with primary trabecular meshwork cells and can be induced to differentiate into phagocytic trabecular meshwork cells in vitro^22^. After being transplanted into wild-type mouse anterior chambers, these stem cells can home to trabecular meshwork tissue and maintain mouse IOP in the normal range^27^.

Other stem cell types have also been explored for trabecular meshwork regeneration. Manuguerra-Gagne et al.^28^ reported that mesenchymal stem cell transplantation rapidly reduced the IOP along with restoration of trabecular meshwork structure 1 month after delivery in rats. Additionally, induced pluripotent stem cells have been differentiated into trabecular meshwork cells in vitro^29^ and restored trabecular meshwork function both ex vivo^30^ and in vivo^31^. Thus both mesenchymal stem cells and induced pluripotent stem cells are potential candidates for cell therapy in glaucoma.

Since TMSCs can differentiate into trabecular meshwork cells by default^22^, TMSCs may be an attractive candidate for cell-based therapy for trabecular meshwork regeneration to control IOP. In this study, we used laser photocoagulation to damage only half of the trabecular meshwork circumference without IOP elevation and report that human TMSCs are able to preferentially home to the laser-damaged mouse trabecular meshwork region after intracameral injection, repair the damaged tissue, and maintain normal IOP, unlike the situation with injected fibroblasts. We also explore possible mechanisms of stem cell homing and repair. Our study shows that stem cell-based therapy is feasible as a new approach to restore glaucomatous trabecular meshwork function.

## Results

### TMSCs home to laser-damaged trabecular meshwork region after intracameral injection

**Fig. 1:**
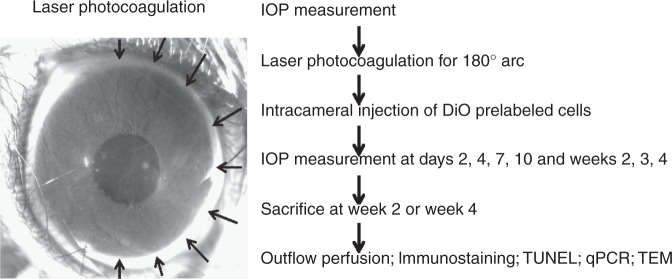
Schematic diagram of the workflow. The photo shows a representative mouse eye that has received laser photocoagulation over a 180° arc (arrows) followed by intracameral injection of cells or sham controls

**Fig. 2:**
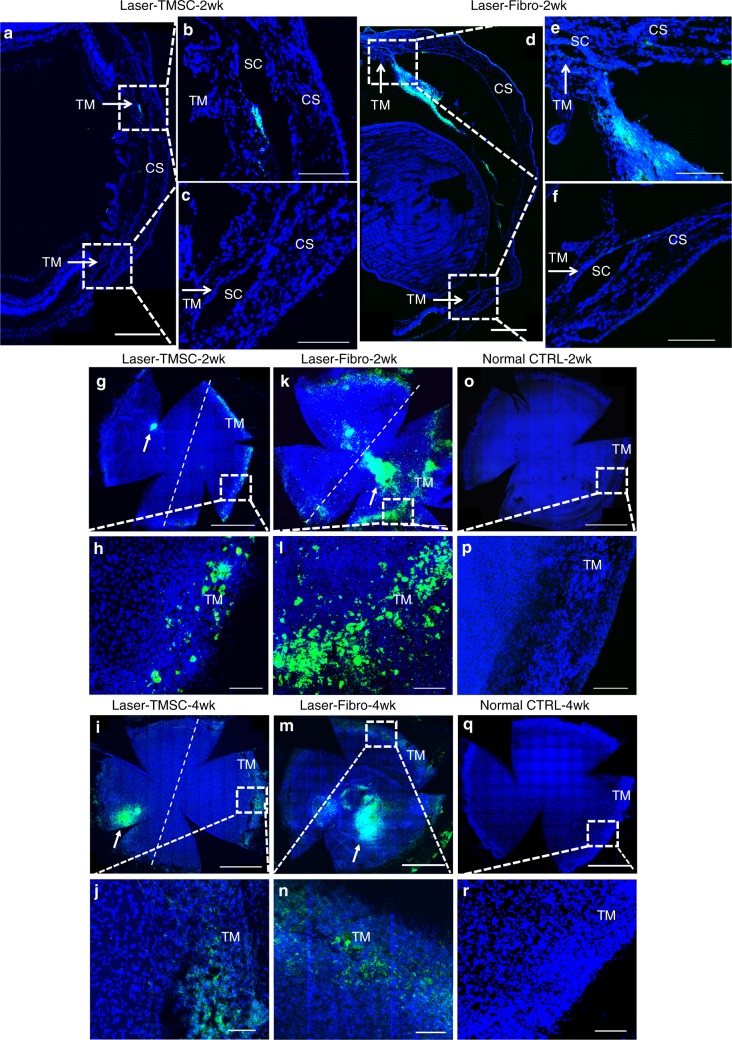
Human TMSCs home to laser-damaged trabecular meshwork region after intracameral injection. TMSCs and fibroblasts were prelabeled with DiO (green) prior to injection and the sections and wholemounts were stained with DAPI (blue). Cryosections (**a**–**f**) show localization of injected TMSCs (**a**–**c**) or fibroblasts (**d**–**f**) 2 weeks after transplantation. Scale bars, 300 μm. **b**, **c**, **e**, **f** are magnifications of the boxed regions in **a**, **e**. **b**, **e** show laser-damaged regions, while **c**, **f** are unlasered regions. Arrows point to the trabecular meshwork. Scale bars, 100 μm. Wholemounts show distribution of transplanted TMSCs at 2 weeks (**g**, **h**) and 4 weeks (**i**, **j**), transplanted fibroblasts at 2 weeks (**k**, **l**) and 4 weeks (**m**, **n**), control eyes without cell transplantation (**o**–**r**). The right side of the dotted line is the laser-damaged region, whereas the left side is unlasered region. **h**, **f**, **p**, **j**, **n**, **r** are magnifications of the boxed regions in **g**, **k**, **o**, **i**, **m**, **q**. The green cell clusters on the corneas where white arrows point to (**g**, **l**, **k**, **m**) were injected cells healing the cornea wounds caused by injection needles. Scale bars, 1 mm (**g**, **k**, **o**, **i**, **m, q**); 100 μm (**h**, **f**, **p**, **j**, **n**, **r**). TM trabecular meshwork, SC Schlemm’s canal, CS corneal stroma

Laser photocoagulation on the trabecular meshwork in C57BL/6 mice was performed over a 180° arc from 6 to 12 o’clock along the limbus (Fig. 1). The laser settings were similar to previously reported^32^ but lasering was only carried out on half of the trabecular meshwork circumference without repetition. Immediately after laser treatment, 50,000 DiO-labeled human TMSCs (Laser-TMSC), human corneal stromal fibroblasts (Laser-Fibro), or media (Laser-Sham) were injected into the mouse anterior chambers. Human TMSCs were isolated and passaged as previously reported^22^. TMSCs from three different donors were used for animal experiments. These cells expressed CD73, CD90, CD105, and CD166 (Supplementary Fig. 1). Using immunofluorescent staining, TMSCs were detected at 2 weeks after transplantation in the mouse trabecular meshwork at sites where laser treatment was received (Fig. 2a, b). Few TMSCs were detected in regions of the trabecular meshwork that did not receive laser treatment (Fig. 2a, c). In contrast, injected fibroblasts were primarily detected in the iris and, to a lesser extent, in the trabecular meshwork (Fig. 2d–f). Wholemount staining also showed that TMSCs were primarily detected in the trabecular meshwork region that received laser photocoagulation at 2 weeks (Fig. 2g, h) and 4 weeks (Fig. 2i, j). Fibroblasts were detected in the trabecular meshwork and the corneal endothelium at 2 weeks (Fig. 2k, l) and 4 weeks (Fig. 2m, n) and were not limited to the laser-treated region. In normal controls, there was no detectable green fluorescent label in mouse trabecular meshwork and cornea (Fig. 2o–r).

### TMSCs integrate into the trabecular meshwork and maintain an open anterior chamber angle

**Fig. 3:**
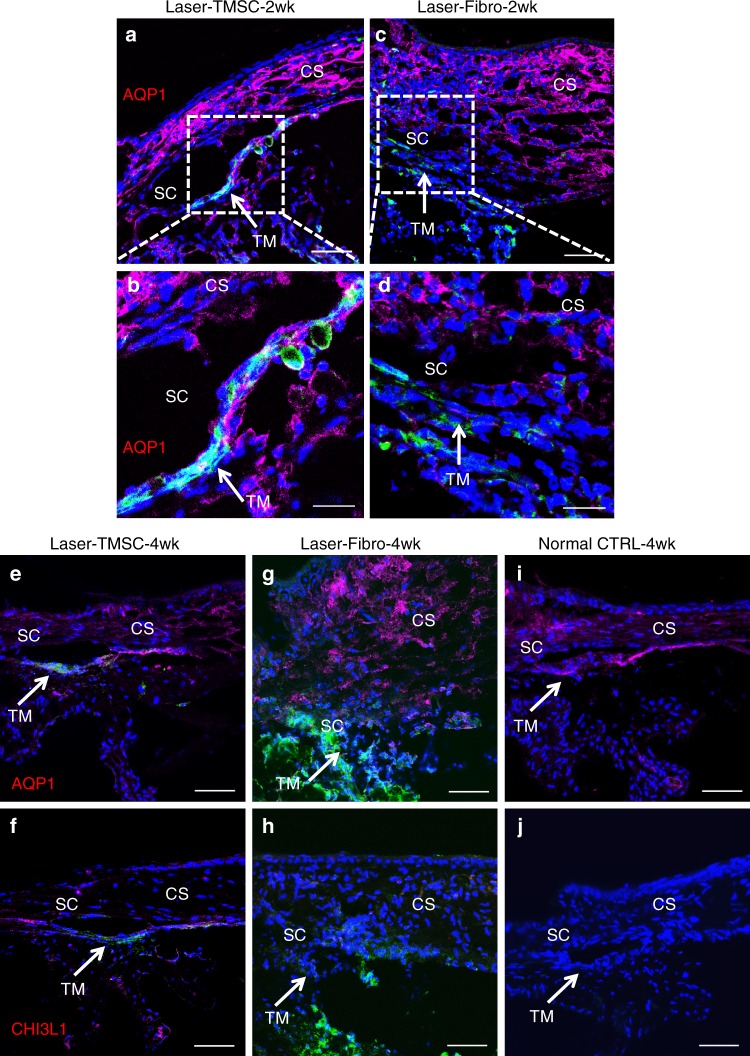
Transplanted TMSCs integrate into the host trabecular meshwork tissue. TMSCs integrated into all layers of the host trabecular meshwork tissue expressing AQP1 (red) as indicated by white arrows at 2 weeks after transplantation (**a**, **b**). Fibroblasts did not integrate into the TM and did not express AQP1 (**c**, **d**). At 4 weeks after transplantation, TMSCs integrated into the TM and expressed AQP1 (**e**) and CHI3L1 (**f**). Fibroblasts did not express AQP1 (**g**) or CHI3L1 (**h**). In non-treated mouse tissue, trabecular meshwork cells, endothelial cells, and keratocytes expressed AQP1 (**i**), whereas none of these mouse cells expressed CHI3L1 (**j**) (note: anti-CHI3L1 is a human-specific antibody and anti-AQP1 antibody recognizes both human and mouse antigens). Arrows point to the TM. Green indicates DiO-labeled (injected) cells. Scale bars, 50 μm (**a**, **c**, **e**–**j**); 20 μm (**b**, **d**). TM trabecular meshwork, SC Schlemm’s canal, CS corneal stroma

Injected TMSCs were distributed throughout all cell layers of the recipient trabecular meshwork at 2 weeks after injection (Fig. 3a, b). The majority of the integrated TMSCs and most of the recipient trabecular meshwork cells were positive for aquaporin 1 (AQP1). None of the injected fibroblasts in the trabecular meshwork tissue expressed AQP1 (Fig. 3c, d). The anterior chamber angle remained open at 2 weeks after TMSC transplantation (Supplementary Fig. 2a, 2b), whereas the eyes with fibroblast injection formed markedly thickened synechia (Supplemental Fig. 2c, d). Similarly, 4 weeks after cell injection, injected human TMSCs were still detectable in all the layers of the trabecular meshwork and expressed the differentiated trabecular meshwork cell markers AQP1 (Fig. 3e) and Chitinase 3-like1 (CHI3L1, Fig. 3f). None of the fibroblasts expressed AQP1 (Fig. 3g) or CHI3L1 (Fig. 3h). The mouse trabecular meshwork cells, corneal endothelial cells, and corneal stromal cells expressed AQP1 (Fig. 3i) but were not detectable with a human-specific CHI3L1 antibody (Fig. 3j).

Cell apoptosis was detected by terminal deoxinucleotidyl transferase-mediated dUTP-fluorescein nick end labeling (TUNEL) staining. Apoptotic cells were found in the trabecular meshwork, cornea, and iris in eyes with medium injection (Laser-Sham) 2 weeks after laser photocoagulation (Supplementary Fig. 3a), but no apoptotic cells were detected at 4 weeks (Supplementary Fig. 3b). In the eyes with TMSC injection, almost no transplanted and resident cells were apoptotic at 2 weeks (Supplemental Fig. 3c) and 4 weeks (Supplementary Fig. 3d). With fibroblast transplantation, apoptosis was detected in both resident and injected cells in the trabecular meshwork at 2 weeks (Supplementary Fig. 3e) and 4 weeks (Supplementary Fig. 3f).

### TMSCs suppress inflammatory response and prevent fibrosis after laser photocoagulation

**Fig. 4:**
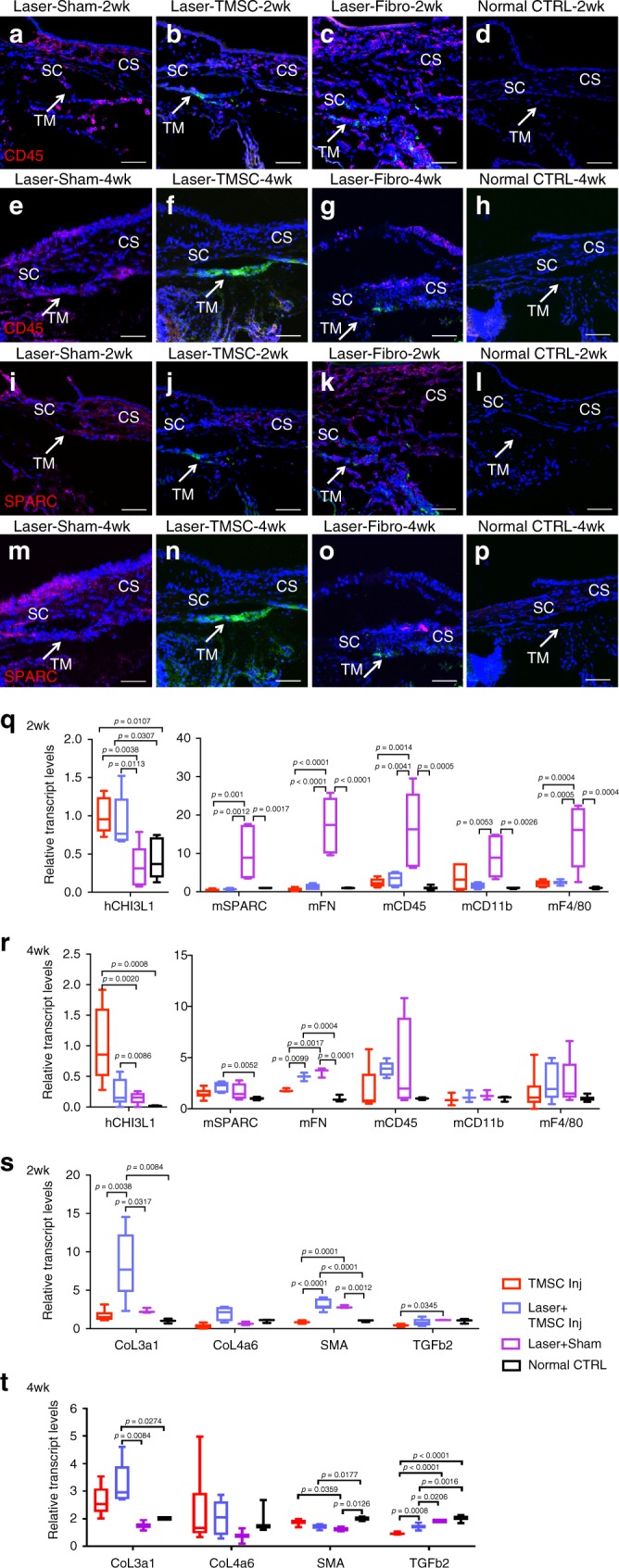
TMSC transplantation strongly attenuates inflammatory and fibrotic responses following laser photocoagulation. Cryosections of normal controls (**a**, **e**, **i**, **m**), lasered eyes with sham injection (**b**, **f**, **j**, **n**), lasered eyes with TMSC injection (**c**, **g**, **k**, **o**), and lasered eyes with fibroblast injection (**d**, **h**, **l**, **p**) were stained with anti-CD45 antibody (red, **a**–**h**) or anti-SPARC antibody (red, **i**–**p**). **a**–**d** and **i**–**l** show the 2-week time point, and **e**–**h** and **m**–**p** show the 4-week time point after laser and cell injection. DAPI stains nuclei blue. Arrows point to the trabecular meshwork. Scale bars, 50 μm. Expression of human CHI3L1, inflammatory markers mCD45, mCD11b, mF4/80, and fibrotic markers mSPARC and mFN in mouse TM tissue at 2 weeks (**q**) and 4 weeks (**r**) was detected by qPCR. Extracellular matrix components Col III, Col IV, αSMA, and TGFβ2 gene expression in mouse TM tissue was also examined by qPCR at 2-week (**s**) and 4-week (**t**) time points after laser and cell transplantation. Error bars show SD of triplicate analysis from three pooled tissues and biological repeats of two individual experiments using different cell strains from two different donors. TM trabecular meshwork, SC Schlemm’s canal, CS corneal stroma

Previously we have demonstrated^32^ that laser photocoagulation induces an inflammatory response, which peaks at 24 h and starts to decline at 1 week. In the current study, we found that laser photocoagulation induced CD45+ cell infiltration in the trabecular meshwork of the laser+sham injection group at 2 weeks (Fig. 4a) and 4 weeks (Fig. 4e). However, in the eyes with laser+TMSC injection, very few CD45+ cells were detected at 2 weeks (Fig. 4b) and 4 weeks (Fig. 4f) in the trabecular meshwork. There were CD45+ cells in the corneal stroma related to laser photocoagulation (Fig. 4b). In the eyes with laser+fibroblast injection, robust CD45+ cell infiltration was detected in the trabecular meshwork region, as well as in the cornea and iris at 2 weeks (Fig. 4c) and 4 weeks (Fig. 4g). There were no inflammatory cells in the trabecular meshwork and cornea of normal eyes (Fig. 4d, h).

We have also demonstrated^32^ that laser photocoagulation induces fibrosis with increased SPARC (secreted protein, acidic and rich in cysteine) expression in the trabecular meshwork starting at 1 week and lasting up to 12 weeks. Here we found that SPARC was expressed in the trabecular meshwork tissue in laser+sham injected eyes at 2 weeks (Fig. 4i) and 4 weeks (Fig. 4m). SPARC expression was not detected at 2 weeks (Fig. 4j) and 4 weeks (Fig. 4n) in the trabecular meshwork tissue in the eyes with laser+TMSC transplantation but was detected in the cornea (Fig. 4j). SPARC expression was detected in the laser+fibroblast injected eyes in the trabecular meshwork and in the cornea and iris at 2 weeks (Fig. 4k) and 4 weeks (Fig. 4o). There was no SPARC expression in the trabecular meshwork, cornea, and iris in normal controls (Fig. 4d, h, i, p).

Quantitative real-time PCR (qPCR) was performed on the mouse trabecular meshwork and the adjacent corneal tissue using primers designed for either human or mouse species. Expression of human CHI3L1 mRNA was detected in the mouse trabecular meshwork with laser+human TMSC transplantation at 2 weeks (Fig. 4q) and 4 weeks (Fig. 4r). Expression of inflammatory markers CD45, CD11b, and F4/80 and fibrotic markers SPARC and fibronectin in the mouse trabecular meshwork with laser+sham injection was dramatically increased at 2 weeks (Fig. 4q). The expression of the above genes in the mouse trabecular meshwork and the adjacent corneal tissue with TMSC injection with or without laser photocoagulation was comparable to those in normal control eyes (Fig. 4q). The increased mRNA expression reduced at 4 weeks after treatment (Fig. 4r). To determine the effects of TMSC transplantation on extracellular matrix (ECM) and trabecular meshwork contractility, gene expression of mouse collagen III (Col III), Col IV, alpha smooth muscle actin (αSMA), and transforming growth factor β2 (TGFβ2) was examined by qPCR on mouse trabecular meshwork and the adjacent corneal tissues. At 2 weeks (Fig. 4s), the expression of Col III and αSMA in the laser+TMSC injection group increased significantly compared to other groups. TMSC injection without laser had decreased expression of TGFβ2 compared to normal and laser controls. TGFβ2 expression in the TMSC injection group reduced compared to laser treatment. At 4 weeks (Fig. 4t), the increased level of Col III in laser+TMSC injection declined but was still significantly higher than that in laser+sham group and normal control. The mRNA expression levels of αSMA and TGFβ2 were different but the changes were less than two folds. The difference of Col IV message expression was not statistically significant at both 2 and 4 weeks.

### TMSCs reconstruct the trabecular meshwork structure after laser photocoagulation

**Fig. 5:**
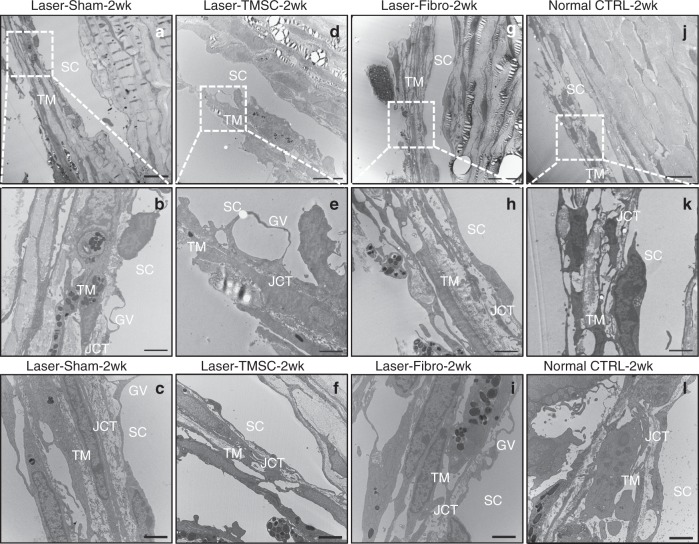
TMSC transplantation restored trabecular meshwork ultrastructure after laser photocoagulation. Transmission electron microscopy (TEM) was performed on mouse trabecular meshwork tissue sections. The regions encased in the boxes (**a**, **d**, **g**, **j**) are magnified in **b**, **e**, **h**, **k**. Normal mouse trabecular meshwork has 3–4 layers of aligned collagen beams covered with cells (**a**–**c**). Giant vacuoles were seen on Schlemm’s canal endothelial cells. The juxtacanalicular connective tissue appeared to contain organized ECM (**a**–**c**). Two weeks after laser and sham injection, the trabecular meshwork had 1–2 layers of disorganized beams (**d**, **e**). Four weeks after laser-Sham, the trabecular meshwork had 2–3 layers of beams and more ECM than in control eyes (**f**). The trabecular meshwork had 3–4 layers of cell-covered beams after laser and TMSC injection at 2 weeks (**g**, **h**) and 4 weeks (**i**) with organized ECM and giant vacuoles (**i**), similar to normal controls (**a**–**c**). With laser and fibroblast injection at 2 weeks (**j**, **k**) and 4 weeks (**l**), TM cells lost nuclei and mitochondria, and the ECM and JCT were disorganized and compacted. Scale bars, 10 μm (**a**, **d**, **g**, **j**), 2 μm (**b**, **e**, **h**, **k**, **c**, **f, i**, **l**). TM trabecular meshwork, SC Schlemm’s canal, JCT juxtacanalicular connective tissue, GV giant vacuoles

Transmission electron microscopy (TEM) (Fig. 5) showed that normal trabecular meshwork had 3–4 cell layers with organized ECM as expected (Fig. 5a–c). Laser-induced trabecular meshwork cell death was evident as loss of nuclei and mitochondria and rupture of cell membranes at 2 weeks (Fig. 5d, e). The damaged trabecular meshwork had partially recovered at 4 weeks (Fig. 5f), consistent with the results of cell apoptosis after laser photocoagulation (Supplementary Fig. 3). TMSC transplantation restored the trabecular meshwork structure at 2 weeks (Fig. 5g, h) and 4 weeks (Fig. 5i). The cells in the restored trabecular meshwork with TMSC transplantation were of normal size and morphology, which were similar to normal controls. Formation of giant vacuoles (Fig. 5i) in Schlemm’s canal endothelial cells indicated that the recovered tissue was functional and allowed aqueous outflow. However, in eyes receiving laser+fibroblast injection, the cells in the trabecular meshwork had condensed nuclei, and the trabecular meshwork itself showed compacted collagen fibrils and disorganized ECM at 2 weeks (Fig. 5j, k) and 4 weeks (Fig. 5l), consistent with fibrosis (Fig. 4o) that would prevent efficient aqueous outflow.

### TMSC transplantation maintains normal IOP and outflow facility after laser photocoagulation

**Fig. 6:**
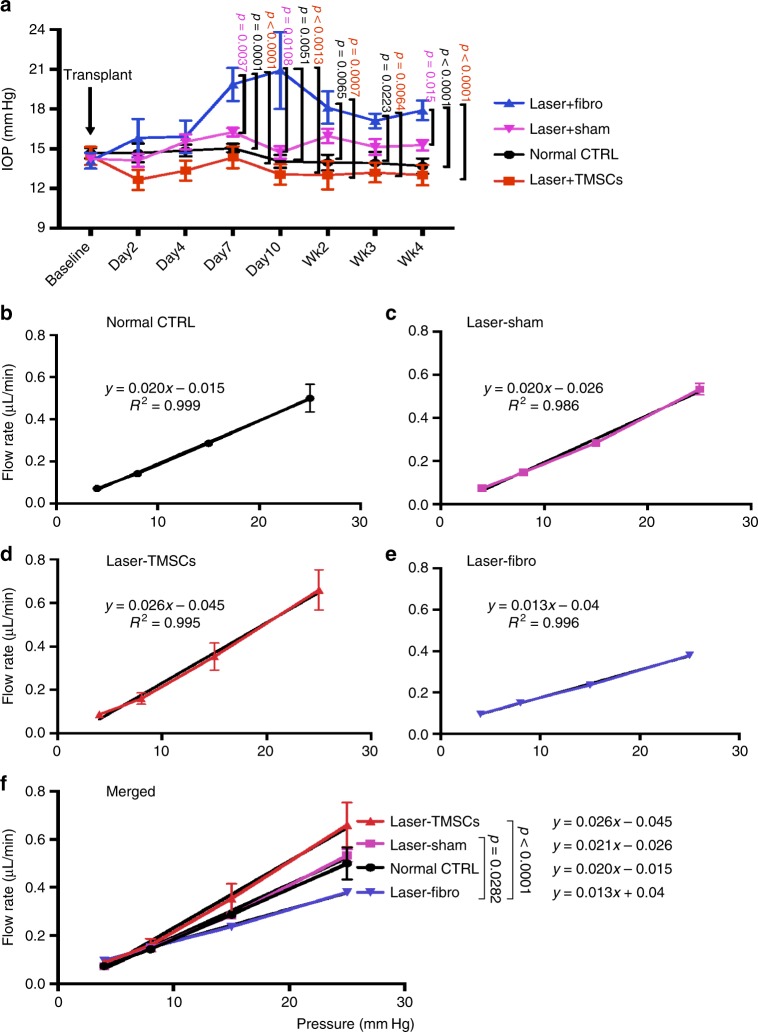
Changes of IOP and aqueous outflow facility after laser and cell transplantation. **a** IOP was measured before all treatments (baseline) and at days 2, 4, 7, and 10 and weeks 2, 3, and 4 post laser and cell transplantation. **b**–**f** Outflow facility was measured by ex vivo eye perfusion at 4-week post laser and cell transplantation. The slope (*y*) of the linear regression line to the flow rate–IOP data represents outflow facility. **b**–**e** shows the individual data and **f** compares the four groups together

It was reported that average IOP, measured by direct ocular puncture, for >30 mouse strains ranged from 10 to 20 mm Hg^33^. Our previous study on 281 C57BL/6 mice showed an average IOP of 14.5 ± 2.6 mm Hg (mean ± SEM), measured by rebound tonometry, with 95th percentile values ranging from 10 to 20 mm Hg^32^. Using the same rebound tonometry IOP measurement system in this study, mice with laser photocoagulation and TMSC injection (Laser-TMSC) had reduced IOP throughout the observation period up to 4 weeks compared to the mice with laser and fibroblast injection (Laser-Fibro, Fig. 6a). Laser-Fibro mice had significantly elevated IOP at day 7 compared to normal control and Laser-TMSC mice, which peaked at day 10 and remained elevated up to 4 weeks. The IOP of Laser-Fibro mice was significantly higher than Laser-Sham controls at days 7 and 10 but this difference was not significant for time points at week 2 and beyond. There were no statistically significant differences in IOP between normal control, Laser-TMSC, and Laser-Sham at every time point measured.

Aqueous outflow facility is inversely proportional to the aqueous flow resistance in the conventional outflow pathway. Mouse eyes were perfused ex vivo to measure outflow facility following the procedures described by Lei et al.^34^, with the slopes of regressed lines (*y*) in Fig. 6b–f representing outflow facility. The outflow facility of Laser-Fibro eyes (Fig. 6e, f) was significantly lower than that of Laser-TMSC eyes (Fig. 6d, f, *p* < 0.0001) and Laser-Sham eyes (Fig. 6c, f, *p* = 0.0282). There were no significant differences in outflow facility between the other groups.

### TMSC homing is associated with the chemokine pair C-X-C chemokine receptor type 4 (CXCR4)/stromal cell-derived factor 1 (SDF1)

**Fig. 7:**
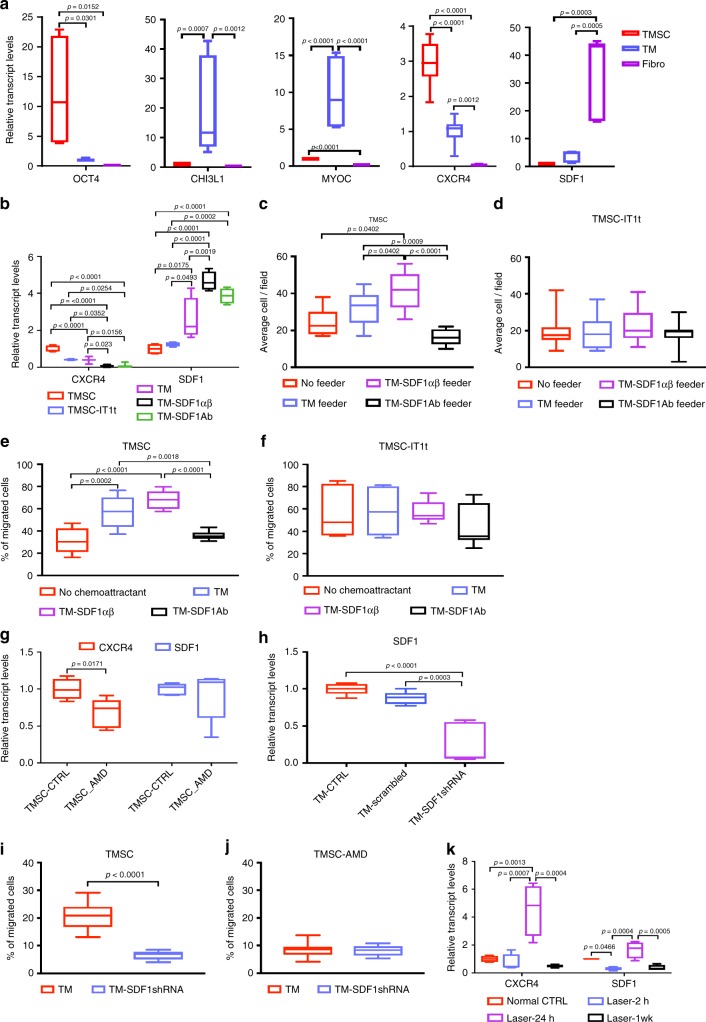
Differential *CXCR4/SDF1* gene expression and affinity and chemotaxis between TMSCs and TM cells. **a** Gene expression in human TMSCs, trabecular meshwork cells, and fibroblasts was compared by qPCR. **b** *CXCR4 and SDF1* gene expression in TMSCs, TMSC-IT1t (TMSCs treated with IT1t), trabecular meshwork cells, TM-SDF1αβ (trabecular meshwork cells treated with SDF1α+1β), or TM-SDF1Ab (trabecular meshwork cells treated with SDF1 antibody) was detected by qPCR. **c** Attached TMSCs or **d** TMSC-IT1t were counted and averaged on different feeder conditions: directly on dishes (No feeder), TM feeder, TM-SDF1αβ feeder, or TM-SDF1Ab feeder. Chemotaxis results are shown as percentage of migrated TMSCs (**e**) or TMSC-IT1t (**f**), defined as the number of migrating cells divided by the sum of migrating and non-migrating cells per view. **g** CXCR4 and SDF1 gene expression in TMSCs treated with AMD3100 was compared with that of TMSCs by qPCR. **h** SDF1 gene expression was compared on TM cells, TM cells treated with scrambled shRNA, and TM cells treated with SDF1 shRNA. Chemotaxis results are shown for TMSCs (**i**) and TMSC-AMD (TMSCs treated with AMD3100) (**j**) with TM cells or TM-SDF1shRNA (TM cells treated with SDF1 shRNA) as chemoattractants. **k** qPCR was performed on mouse trabecular meshwork tissue and adjacent corneal tissue after laser photocoagulation at 2 h, 24 h, and 1 week to compare CXCR4/SDF1 expression with normal control

It has been reported that chemokine SDF1 (also called CXCL12) and its receptor CXCR4, which together constitute the CXCR4/SDF1 axis, play important roles in hematopoietic stem cell homing^35^. In order to investigate whether the CXCR4/SDF1 axis plays a role in TMSC homing to damaged trabecular meshwork tissue, we first evaluated gene expression in TMSCs and trabecular meshwork cells. As shown in Fig. 7a, TMSCs had higher expression of OCT4 and CXCR4 and lower expression of CHI3L1, MYOC, and SDF1 than trabecular meshwork cells. SDF1 expression in fibroblasts was statistically higher compared to both trabecular meshwork cells and TMSCs. To examine the affinity between different cell types, we seeded trabecular meshwork cells as feeders for 24 h as illustrated in Supplementary Fig. 4a. DiO-labeled TMSCs, trabecular meshwork cells, or fibroblasts were then added to wells with or without feeder cells. At 30 and 60 min after seeding, the wells were washed and fixed. Attached DiO+ cells were counted after imaging (Supplementary Fig. 5a-5d). The numbers of TMSCs attached to the wells with trabecular meshwork feeders at both time points (9.4 ± 3.4 cells/field at 30 min, 18.9 ± 8.9 cells/field at 60 min, Supplementary Fig. 5e, 5f) were significantly greater than the numbers at other conditions (*p* < 0.0001).

To confirm that the CXCR4/SDF1 chemokine axis is involved in TMSC and trabecular meshwork cell interaction, we treated TMSCs with the CXCR4 inhibitor IT1t^36^ (TMSC-IT1t) for 72 h to reduce CXCR4 expression on TMSCs. qPCR showed that CXCR4 expression in TMSC-IT1t cells was reduced by approximately 60% compared to untreated TMSCs (Fig. 7b), similar to levels of trabecular meshwork cells. We also cultured trabecular meshwork cells with recombinant human SDF1α and 1β for 72 h to increase the SDF1 expression (TM-SDF1αβ) or with anti-SDF1 antibody for neutralization of SDF1 in the trabecular meshwork cells (TM-SDF1Ab). The SDF1 expression on TM-SDF1αβ cells increased by 20% compared to trabecular meshwork cells. In contrast, the SDF1 expression on TM-SDF1Ab cells was reduced to 25% of that in untreated trabecular meshwork cells (Fig. 7b).

We then evaluated cell affinity between TMSCs and trabecular meshwork cells with natural or modified CXCR4 or SDF1 expression. DiO-labeled TMSCs or TMSC-IT1t cells were seeded directly on culture dishes or dishes preseeded with trabecular meshwork, TM-SDF1αβ, or TM-SDF1Ab cells as illustrated in Supplemental Fig. 4a. At 60 min, the dishes were washed, imaged, and DiO-labeled cells were counted (Supplementary Fig. 5). At least five fields of each condition were imaged, counted, and averaged. The experiment was repeated once with TMSCs and trabecular meshwork cells from different donors. Figure 7c shows the average numbers of attached TMSCs per field in each condition with different feeders and Fig. 7d shows attached numbers of TMSC-IT1t cells. The number of attached TMSCs on TM-SDF1αβ feeders was the greatest (41.8 ± 9.9 cells/field), while the number of TMSCs on TM-SDF1Ab feeders was the least (16.5 ± 4.4 cells/field). Differences in TMSC cell counts on different feeders were statistically significant (*p* = 0.042–*p* < 0.0001, Fig. 7c). After TMSCs were treated with IT1t, the attached cell numbers for the different feeder conditions were similar, with no statistically significant differences between groups (*p* > 0.05, Fig. 7d).

In addition to affinity assay, the effects of CXCR4/SDF1 on chemotaxis between TMSCs and trabecular meshwork cells without direct contact were also examined, as illustrated in Supplemental Fig. 4b. Untreated trabecular meshwork cells, TM-SDF1αβ, or TM-SDF1Ab cells were seeded on the bottom of cell culture wells to allow attachment for 24 h. DiO-labeled TMSCs or TMSC-IT1t were seeded on Transwell membranes with 8-μm pore size for 24 h. Non-migrating cells (those remaining on the seeded side, Supplementary Fig. 7a-d, 7i-l) and cells that migrated to the other side of the membrane (Supplementary Fig. 7e-h, 7m-p) were counted in five randomly selected microscopic fields at ×20 magnification. The experiment was repeated with cells from two donors. The migration rate of TMSCs toward SDF1αβ trabecular meshwork cells in the bottom chamber was significantly greater than that when no cells were present in the bottom chamber (medium only) (57.1 ± 14.9% vs. 31.3 ± 11.3%, *p* < 0.0001, Fig. 7e). With SDF1Ab trabecular meshwork cells in the bottom chamber, the percentage of TMSCs migrating was reduced to 35.9 ± 3.9% (*p* = 0.0018 compared to trabecular meshwork cells at the bottom; *p* < 0.0001 compared to SDF1αβ trabecular meshwork cells at the bottom, Fig. 7e). There was no difference between TMSC migration with trabecular meshwork cells in the bottom chamber (47.1 ± 14.9%) vs. with SDF1αβ trabecular meshwork cells (68.2 ± 8.2%) (*p* > 0.05, Fig. 7e) in the bottom chamber. Similar to the cell attachment results, the difference of migration rates of TMSC-IT1t for different cell feeder conditions was not statistically significant (*p* > 0.05, Fig. 7f).

To further confirm CXCR4/SDF1 effects on chemotaxis, TMSCs were also treated with another CXCR4 antagonist, AMD3100, to reduce CXCR4 expression. Figure 7g shows that CXCR4 expression was reduced about 1/3 of that in untreated TMSCs as confirmed by qPCR, while SDF1 expression was not affected (Fig. 7g). Trabecular meshwork cells were also treated with short hairpin RNA (shRNA) to reduce SDF1 expression to 30% of that in trabecular meshwork cells (Fig. 7h). The cell migration rate of TMSCs with TM-SDF1shRNA on the bottom as a chemoattractant was significantly reduced (6.6 ± 1.5) compared to that with trabecular meshwork cells as a chemoattractant (20.8 ± 4.8, Fig. 7i). After TMSCs were treated with AMD3100 (TMSC-AMD), the migration rates were similar (Fig. 7j) with either trabecular meshwork cells (8.7 ± 2.8) or TM-SDF1shRNA (8.1 ± 2.0, Fig. 7j) as chemoattractants. The experiment was repeated with cells from two different donors.

We also examined CXCR4/SDF1 expression in mouse trabecular meshwork tissue with and without laser photocoagulation. Mouse trabecular meshwork with adjacent corneal tissue was collected and three tissues were pooled per condition for RNA extraction, with results averaged from at least two biological repeats. Expression of CXCR4 in mouse trabecular meshwork 24 h after laser photocoagulation increased about five-fold compared to normal controls and then declined to the normal control level at 1 week (Fig. 7k). Similarly, expression of SDF1 in mouse trabecular meshwork 24 h after laser photocoagulation increased about two-fold compared to normal controls and declined to the normal control level at 1 week (Fig. 7k). Both increases were statistically significant compared to all the other groups.

## Discussion

In this study, we developed a new mouse model wherein a half circumference of trabecular meshwork tissue was injured by laser photocoagulation, allowing us to test the ability of TMSCs to home to damaged trabecular meshwork tissue and restore the structure and function of the trabecular meshwork. We note that it is not an ocular hypertension model. Importantly, our data show that TMSCs preferentially home to the laser-damaged trabecular meshwork region and integrate into the trabecular meshwork tissue after intracameral injection. Some integrated TMSCs expressed the differentiated trabecular meshwork cell markers AQP1 and CHI3L1. The eyes receiving TMSC transplantation maintained normal IOP and normal outflow facility, and their anterior chamber angles remained open. In contrast, fibroblasts appeared to passively follow the aqueous outflow and resided on the trabecular meshwork region or attached to the cornea and iris non-specifically; further, the anterior chamber angle demonstrated thickened synechia. The inflammatory and fibrotic response induced by laser photocoagulation was dramatically reduced with TMSC transplantation and exaggerated by fibroblast transplantation. The laser-damaged trabecular meshwork ultrastructure was regenerated after TMSC transplantation, as shown by TEM. Furthermore, we showed that TMSCs and trabecular meshwork cells have differential expression of the chemokine pair CXCR4/SDF1 and that this chemokine pair plays an important role in the affinity and chemotaxis between TMSCs and trabecular meshwork cells in vitro and likely plays a role in TMSC homing in vivo. Our data show that TMSC transplantation maintains normal aqueous outflow in this model and restores trabecular meshwork structure while attenuating fibrosis and inflammation; since fibrosis and inflammation have been proposed to play a role in trabecular meshwork dysfunction in certain types of glaucoma^37–39^, our data suggest that TMSCs could be a novel glaucoma treatment targeted at the conventional outflow pathway to reduce IOP and thereby prevent optic nerve damage and vision loss.

AQP1 has been detected in the trabecular meshwork in vivo^27,40^ as well as in cultured human trabecular meshwork cells^22,41^ and plays an important role in the modulation of aqueous outflow^41^. CHI3L1 is involved in tissue remodeling and is considered as a differentiation marker of trabecular meshwork cells^18,22,42–44^. The expression of AQP1 and CHI3L1 in the transplanted TMSCs and these transplanted cells were detected in all 3–4 layers of the mouse trabecular meshwork tissue (Fig. 3), indicating that TMSCs integrated into the trabecular meshwork and differentiated to functional trabecular meshwork cells and were able to remodel the trabecular meshwork and maintain normal aqueous outflow. Further, since only a proportion of the transplanted TMSCs differentiated into trabecular meshwork cells, we suspect that the remaining stem cells maintained their undifferentiated state in vivo and could serve as a cell source for ongoing trabecular meshwork regeneration.

In mammals, the regenerative capacities of endogenous stem cells decrease progressively with age^45^. It is reported that hematopoietic stem cells were predisposed to apoptosis in response to endoplasmic reticulum stress^46^, which may lead to exhaustion of the in situ stem cell pool. Endogenous stem cell regeneration is a complex process, including cell proliferation, differentiation, and migration, that requires cues from stem cell niches, chemokines, and other factors. Mobilization of endogenous stem cells for tissue repair and regeneration is being explored^47,48^. Additionally, transplantation of exogenous stem cells might affect activation of endogenous stem cells, which needs to be further explored.

We have shown that laser photocoagulation induces elevated IOP^32^ after 2–3 rounds of laser treatment. In this study, we carried out only one round of laser photocoagulation to damage only half of the circumference of the trabecular meshwork and thus did not elevate IOP. We found that TMSC transplantation can slightly lower IOP and increase outflow facility, but both parameters remained in their normal ranges. However, transplantation of human fibroblasts induced a significant drop in outflow facility with a corresponding IOP elevation. These results indicate that xenotransplantation of human TMSCs has the ability to maintain trabecular meshwork function in mice.

Xenotransplantated human TMSCs not only survived up to 4 weeks but also prevented host trabecular meshwork cell death after laser photocoagulation (Supplementary Fig. 3). TMSCs suppressed the inflammatory and fibrotic response by dramatically reducing CD45+ inflammatory cell infiltration including monocytes (CD11b+) and macrophages (F4/80+) and reduced the expression of SPARC and fibronectin (Fig. 4). The matricellular protein SPARC is associated with excessive wound healing, increased fibrosis, and scar formation in glaucoma pathogenesis^3,32,49^. Fibronectin induces collagen deposition and is elevated in glaucomatous trabecular meshwork and aqueous humor^50,51^. Our previously published results also proved that xenotransplantation of human corneal stromal stem cells^52^ and human TMSCs^27^ did not elicit inflammatory response or immunorejection.

ECM in the trabecular meshwork tissue plays important roles in regulating aqueous humor outflow and thus in controlling IOP. The cytokine TGFβ2 has an important role in the trabecular meshwork, inducing expression of collagens, αSMA, and fibronectin^53^. Our results showed that laser photocoagulation increased the expression of αSMA in the trabecular meshwork tissue at 2 weeks, while laser together with TMSC injection increased the expression of αSMA and Col III (Fig. 4s). These increases declined to normal level at 4 weeks, except Col III expression in laser+TMSC injection was still increased. This data may indicate that TMSC transplantation can promote ECM turnover and have effects on trabecular meshwork homeostasis. Further studies are needed to elucidate how TMSCs regulate ECM and respond to TGFβ2 stimulation.

We have shown that TMSCs home to the normal mouse trabecular meshwork after transplantation^27^. Here we further show that transplanted TMSCs preferentially home and integrate into the partially damaged trabecular meshwork, with very few reaching the normal undamaged trabecular meshwork (Fig. 2). It has been reported that chemokines, notably CXCR4/SDF1, are involved in stem cell homing^35^. Many other chemokines have been shown to be involved in mesenchymal stem cell homing to injured sites^54^. Our results demonstrate that passaged human TMSCs have higher expression of CXCR4, while differentiated trabecular meshwork cells and fibroblasts express higher levels of SDF1; expression of CXCR4 and SDF1 in mouse trabecular meshwork tissue dramatically increased 24 h after laser and reduced to normal range at 1 week. This indicates that laser photocoagulation might have activated the proliferation and migration of both TMSCs and trabecular meshwork cells in vivo. Our in vitro study showed that the affinity and chemotaxis between TMSCs with trabecular meshwork cells or SDF1αβ trabecular meshwork cells were statistically higher than TMSCs with trabecular meshwork cells with SDF1 neutralized by incubation with SDF1 antibody (TM-SDF1Ab). When CXCR4 expression in TMSCs was reduced by treatment with the CXCR4 antagonist IT1t, there was no statistical difference of the affinity and chemotaxis between the groups. We further used another CXCR4 antagonist, AMD3100, to reduce CXCR4 expression in TMSCs and transfected SDF1 shRNA into trabecular meshwork cells to reduce SDF1 expression in trabecular meshwork cells, confirming the effects of the CXCR4/SDF1 axis on chemotaxis between TMSCs and trabecular meshwork cells. Although increasing SDF1 expression in the trabecular meshwork cells did not increase the affinity and chemotaxis between TMSCs and trabecular meshwork cells, reduction of SDF1 or neutralization of SDF1 in trabecular meshwork cells and reduction of CXCR4 in TMSCs did reduce the affinity and chemotaxis between TMSCs and trabecular meshwork cells. Further studies using CRISPR/Cas9 technology to more efficiently block CXCR4 expression or increased CXCR4 expression in TMSCs may be needed to confirm our current results. Moreover, cell homing is multifactorial and other factors such as integrins and cadherins are also likely involved in this process. Further studies are needed to fully elucidate TMSC-homing mechanisms.

Understanding stem cell-homing mechanisms is critical to enhance homing and integration of transplanted stem cells and to better restore injured trabecular meshwork function for future clinical applications. Our study provides compelling evidence for the involvement of the CXCR4/SDF1 axis as an important mechanism for TMSC homing. Further studies are needed to elucidate how stem cells reconstruct the trabecular meshwork tissue and how integrated cells modulate outflow facility.

The successful TMSC xenotransplantation we here report in mouse suggests the possibility of allotransplantation of TMSCs in human patients. It will be essential to build up human TMSC banks first and then do allotransplantations in larger animals, including non-human primates, before eventually carrying out TMSC transplantations in human glaucoma patients.

## Methods

### Cell culture and passage

Human TMSCs were cultured and passaged similar to what described previously^22^. Deidentified human corneas not usable for transplantation were obtained from the Center for Organ Recovery and Education (Pittsburgh, PA). The TM tissue was carefully peeled off after removing the iris and ciliary process and a cut along the anterior margin of the Schwalbe’s line. The tissue was cut into quarters and put in a 25-cm^2^ culture flask for 10–14 days in a culture medium containing Opti-MEM (Invitrogen) supplemented with 5% fetal bovine serum (FBS; ThermoFisher), 10 ng/ml epidermal growth factor (Sigma), 100 μg/ml bovine pituitary extract (Life Technologies), 20 μg/ml ascorbic acid, 200 μg/ml calcium chloride, 0.08% chondroitin sulfate (Sigma-Aldrich), 100 IU/ml penicillin, 100 μg/ml streptomycin, and 50 μg/ml gentamicin (ThermoFisher)). After trypsinization with Tryple (Invitrogen), the cells were seeded at 500–1000 cells/cm^2^ to allow clonal growth and passaged at 70–80% confluence. Human trabecular meshwork cells were cultured in the similar explant culture to TMSCs in Dulbecco’s modified Eagle’s medium (DMEM)/F12 with 10% FBS and were passaged when cells reached 100% confluence. Human fibroblasts were cultured and passaged from human corneal stroma as previously described^27^. In brief, human corneal stroma was digested with collagenase at 0.5 mg/ml at 37 °C overnight and washed with DMEM/F12 three times. The cells were cultured in DMEM/F12 with 10% FBS and passaged after completely confluent.

### Laser photocoagulation and transplantation of human cells into mouse anterior chamber

Healthy female and male adult C57BL/6 mice aged 8 weeks were purchased from Charles River Laboratories. Mice were maintained in the University of Pittsburgh Animal Facility with a 12-h light–dark cycle and free access to food and water. All experimental procedures were reviewed and approved by the University of Pittsburgh Institutional Animal Care and Use Committee and animals were handled according to guidelines provided in the Association for Research in Vision and Ophthalmology Resolution on the Use of Animals in Ophthalmic and Vision Research.

Laser photocoagulation was performed on 9–11-week-old C57BL/6 mice (both male and female) following the method previously described^32^ with modifications. In brief, a 532 nm cw-diode laser (IRIDEX) was used with 80-mW laser power, 150-ms duration, and 50-μm diameter spot size. Mice were anesthetized by intraperitoneal injection of ketamine hydrochloride (50 mg/kg) and xylazine (0.5 mg/kg) (IVX Animal Health,) and each eye typically received 9–11 laser spots over an 180° arc of the trabecular meshwork without repetition. Right eyes of mice were treated. Immediately after laser photocoagulation, passage 3 or 4 of TMSCs, passage 6 or 7 of fibroblasts, or DMEM/F12 (Sham control) were used for cell transplantation. Cells were prelabeled with the membrane dye DiO (Invitrogen) to allow tracing as previously described^27,52,55^. Cells were suspended in DMEM/F12 at 1 × 10^6^ cells/ml and DiO was added at a dilution of 50 μg/ml at 37 °C for 30 min. After washes, cells were resuspended in DMEM/F12 at 2.5 × 10^4^ cells/μl for in vivo transplantation. Cell transplantation followed the procedures described previously^27^. In brief, a 30-gauge needle was used to make a corneal tunnel and 1.5 μl air was injected to fill up the anterior chamber with a 10-μl microsyringe fitted with a 33-gauge beveled needle (Hamilton, Reno, NV). Then 2 μl of cells were injected into the anterior chamber with the air bubble blocking the corneal tunnel to prevent cell leakage. Mice were sacrificed at 2 or 4 weeks after injection. IOP was measured under anesthesia by intraperitoneal injection of ketamine and xylazine^27,32^ using a rebound tonometer for rodents (TonoLab) as soon as animals entered sedation. IOP was measured before laser as a baseline and then measured at days 2, 4, 7, and 10 and weeks 2, 3, and 4 between 1 p.m and 4 p.m.

For each set of experiments, there were at least ten mice of each group at each time point. The experiment was repeated twice with TMSCs and fibroblasts from different donors. Additional experiments for qPCR were performed with two different sets of TMSCs from different donors. RNA was extracted from three trabecular meshwork tissues for each condition and results were averaged from two independent experiments. Perfusion on mouse eyes to measure outflow facility was performed on 3–5 eyes of each condition at the 4-week time point.

### Measurement of aqueous humor outflow facility

Outflow facility measurement was performed on enucleated mouse eyes following procedures described by Lei et al.^34^. with minor modifications. The perfusion system consists of a computer-controlled syringe pump (Harvard Apparatus, Hilliston, MA). The anterior chamber was cannulated with a microinjection glass pipette connected to a pressure transducer (Honeywell, Ft. Washington, PA). The pump, loaded with a 25-µl Hamilton syringe filled with phosphate-buffered saline (PBS), delivered a variable flow rate (*Q*) to the anterior chamber to maintain a desired IOP, which was monitored by the pressure transducer that connected to a computer control system (Labview software; National Instruments, Austin, TX). Eyes were perfused with PBS at constant pressures of 4, 8, 15, and 25 mmHg sequentially. At each pressure, the steady pressure was maintained for at least 15 min and the resulting flow rate was averaged. Facility was calculated using the Goldmann equation^34,56^ shown as the slope of the flow-pressure regression line. Data from a given eye were only accepted when *R*^2^ > 0.95.

### Histology

Enucleated mouse eyes were fixed in 1% paraformaldehyde at 4 °C overnight followed by either storage at 4 °C in 50% glycerol and 50% PBS (v/v) for wholemount staining or frozen at −20°C in optimal cutting temperature embedding compound (Electron Microscopy Sciences) and cut into 6-μm thick cryosections for immunofluorescent staining. Nonspecific binding was blocked with 10% heat-inactivated goat serum or 1% bovine serum albumin and anti-mouse CD16/CD32 Fcγ III/II. Primary antibodies were incubated overnight at 4 °C followed by secondary antibodies and DAPI (Invitrogen) incubation for 2 h at room temperature. Images were acquired on a confocal microscope with a ×40 or ×60 oil objective (Olympus FluoView) and analyzed on FV10-ASW4.2 Viewer (Olympus). Antibodies used are shown in Supplementary Table 1.

### Wholemount stain

After fixation, the posterior part of eyes was cut off from 1.5 mm of the limbus. The anterior part of the eyes including the cornea and trabecular meshwork was cut into quarters after carefully removing the lens, iris, and ciliary body. The tissues were incubated with DAPI at room temperature for 30 min and washed five times and mounted for confocal imaging. Stitched image stacks were acquired by sequential scanning on a confocal microscope and analyzed on FV10 viewer.

### TUNEL assay

TUNEL assay was performed using an in situ Cell Death Detection Kit (Roche) following the manufacturer’s protocol on cryopreserved tissue. Nuclei were stained with DAPI. At least three independent eyes from each condition and eight sections of each condition were stained and imaged using a confocal microscope.

### Transmission electron microscopy

The ultrastructure of mouse trabecular meshwork was examined by TEM followed the procedures described previously^32^. Mouse eyeballs were fixed in cold 2.5% glutaraldehyde (EM were then post-fixed in 1% osmium tetroxide (Electron Microscopy Sciences) with 1% potassium ferricyanide (Fisher). They were dehydrated through a graded series of ethanol baths and embedded in Epon (made from dodecenyl succinic anhydride, nadic methyl anhydride, Scipoxy 812 Resin, and 2,4,6-tris(dimethylaminomethyl) phenol (Energy Beam Sciences)). Semi-thin (300 nm) sections were cut on a Reichart Ultracut, stained with 0.5% toluidine blue (Fisher), and examined under a light microscope. Ultrathin sections (65 nm) stained with uranyl acetate (Electron Microscopy Sciences) and Reynold’s lead citrate (Fisher) were examined and photographed at 80 kV on a Jeol 1011 TEM.

### Cell migration assay—chemotaxis

Chemotaxis of trabecular meshwork cells to TMSCs was evaluated by cell migration assay on a Transwell cell culture system. Trabecular meshwork cells were treated with both SDF1α and SDF1β (Millipore) at 80 ng/ml each (TM-SDF1αβ) for 72 h to increase the SDF1 expression in cells. To block SDF1 in the trabecular meshwork cells, they were treated with SDF1 antibody (Millipore, MABC184, azide free) at 1 μg/ml (TM-SDF1Ab) for 72 h to neutralize SDF1 in the cells. Untreated trabecular meshwork cells, TM-SDF1αβ, and TM-SDF1Ab cells were seeded at the bottom of 24-well culture plates as chemoattractant. To reduce CXCR4 expression, TMSCs were treated with CXCR4 antagonist II IT1t (Calbiochem) at 44 nM (TMSC-IT1t) or antagonist AMD3100 (Millipore) at 5 μg/ml (TMSC-AMD) for 72 h. TMSCs, TMSC-IT1t, or TMSC-AMD were labeled with DiO and seeded in the cell culture inserts with 8-μm pores (Costar). After 24-h incubation, the Transwell membranes of the culture inserts were fixed, stained with DAPI, and mounted. Cells migrated to the other side of the membrane and non-migrated cells were counted in five randomly selected microscopic fields at ×20 magnification. Percentage of migrated cells was calculated as the migrated cell number divided by total cell numbers. The treated cells were also lysed with RLT for RNA isolation and qPCR to confirm the gene expression levels.

### Cell affinity assay

Cell affinity assay was performed in eight-chamber glass slides seeded with untreated and treated TM cells (TM-SDF1αβ, TM-SDF1Ab) as described in the “Cell migration assay—chemotaxis” section. Twenty-four hour later, TMSCs, TMSC-IT1t, or TMSC-AMD were labeled with DiO and added into the chambers on top of the feeder cells for 60 min. The slides were washed with PBS twice to remove any unattached cells, fixed, and stained with DAPI and imaged. DiO+ attached cells were counted and compared.

### shRNA treatment

shRNA plasmids to human SDF1 and scrambled shRNA plasmids were purchased from Santa Cruz Biotechnology and transfection followed the procedures described by the manufacturer. Transfected trabecular meshwork cells (TM-SDF1shRNA) were selected by treatment with puromycin to kill non-transfected cells. The reduced SDF1 expression was confirmed by qPCR. Untreated trabecular meshwork cells and TM-SDF1shRNA cells were seeded at the bottom of 24-well culture plates as chemoattractant for cell migration assay.

### Quantitative RT-PCR

qPCR was performed on limbal tissues containing the trabecular meshwork and cornea. The tissue stripes were lysed with RLT buffer in an RNA purification kit (RNeasy Mini Kit, Qiagen) and homogenized with steel beads. RNA was isolated following the manufacturer’s instructions. cDNAs were transcribed using XLAScript cDNA MasterMix (WorldWide). qPCR was performed by direct dye binding (SYBR Green, Applied Biosystems) as previously described^57^. Primers were designed using Primer3 except mouse CD45 and CD11b, which were designed following the description by Wu et al.^58^. Primers were blasted on the NIH website (https://www.ncbi.nlm.nih.gov/tools/primer-blast/) to confirm specificity for human or mouse species. Primers of CHI3L1 were specific for human and primers of CD45, CD11b, F4/80, SPARC, FN, Col3α1, Col4α6, αSMA, and TGFβ2 were specific for mouse. The human SDF1 primer set recognizes human SDF1 mRNA transcript variants 2 and 5. The mouse SDF1 primer set recognizes mouse SDF1 mRNA transcript variants 1, 2, 3, X1, and X2. The sequences are shown in Table S2.

RNA content was normalized by 18S rRNA. Relative mRNA abundance was calculated as the Ct for amplification of a gene-specific cDNA minus the average Ct for 18S expressed as a power of 2 (2^–ΔΔCt^). Three individual gene-specific values thus calculated were averaged to obtain mean ± SD.

### Statistical analysis

All values are presented as mean ± SD, except IOP which is presented as mean ± SEM. Statistical differences, except for outflow facility data, were determined by two-way analysis of variance, followed by the Tukey post test to assess the significance of differences between all groups. For outflow facility data, multiple comparisons of the slopes for the linear regressions among different groups were examined using the generalized linear mixed model (SAS software). *p* Values for multiple comparisons were adjusted by the Bonferroni method. Statistical significance was set at *p* < 0.05.

## Electronic supplementary material

Description of Additional Supplementary Files

Supplementary Information

Supplementary Data 1

Supplementary Data 2

## Data Availability

Data generated by this study are included in the main text and Supplementary Files, including Supplementary Data 1 and 2 and are available from the corresponding authors upon reasonable request.
