# Publisher Correction: Human stem cells home to and repair laser-damaged trabecular meshwork in a mouse model

**DOI:** 10.1038/s42003-021-01958-w

**Published:** 2021-04-06

**Authors:** Hongmin Yun, Yiwen Wang, Yi Zhou, Ke Wang, Ming Sun, Donna B. Stolz, Xiaobo Xia, C. Ross Ethier, Yiqin Du

**Affiliations:** 1 Department of Ophthalmology, University of Pittsburgh, Pittsburgh, PA 15213 USA; 2 Department of Ophthalmology, Xiangya Hospital, Central South University, 410008 Changsha, Hunan China; 3 Department of Biomedical Engineering, Georgia Institute of Technology/Emory University, Atlanta, GA 30332 USA; 4 Department of Cell Biology, University of Pittsburgh, Pittsburgh, PA 15213 USA; 5 McGowan Institute for Regenerative Medicine, University of Pittsburgh, Pittsburgh, PA 15213 USA; 6 Department of Developmental Biology, University of Pittsburgh, Pittsburgh, PA 15213 USA

**Keywords:** Mesenchymal stem cells, Glaucoma

Correction to: *Communications Biology* 10.1038/s42003-018-0227-z, published online 6 December 2018.

In the original version of the Article, errors were introduced in the labels in Fig. 5.

**Figure:**
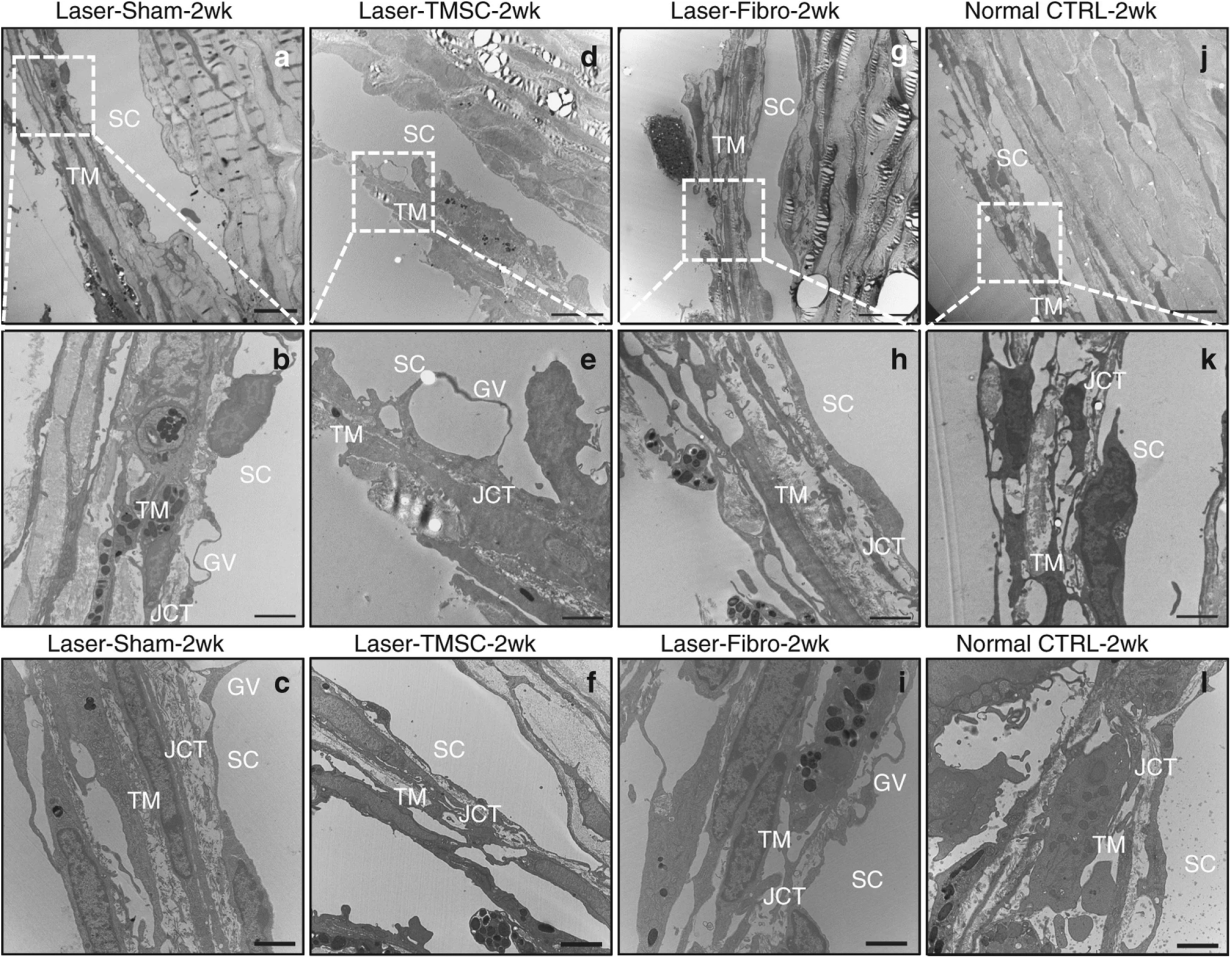
Fig. 5

Panels a–c were incorrectly labelled “Laser-Sham-2wk”. The correct label for panels a and b is “Normal CTRL-2wk”. The correct label for panel c is “Normal CTRL-4wk”.

Panels d–f were incorrectly labelled “Laser-TMSC-2wk”. The correct label for panels d and e is “Laser-Sham-2wk”. The correct label for panel f is “Laser-Sham-4wk”.

Panels g– i were incorrectly labelled “Laser-Fibro-2wk”. The correct label for panels g and h is “Laser-TMSC-2wk”. The correct label for panel i is “Laser-TMSC-4wk”.

Panels j–l were incorrectly labelled “Normal CTRL-2wk”. The correct label for panels j and k is “Laser-Fibro-2wk”. The correct label for panel l is “Laser-Fibro-4wk”.

The originally published (incorrect) Fig. 5 is shown below.

The errors have been corrected in the HTML and PDF versions of the Article.

