# Author Correction: Human stem cells home to and repair laser-damaged trabecular meshwork in a mouse model

**DOI:** 10.1038/s42003-021-01957-x

**Published:** 2021-04-06

**Authors:** Hongmin Yun, Yiwen Wang, Yi Zhou, Ajay Kumar, Ke Wang, Ming Sun, Donna B. Stolz, Xiaobo Xia, C. Ross Ethier, Yiqin Du

**Affiliations:** 1 Department of Ophthalmology, University of Pittsburgh, Pittsburgh, PA 15213 USA; 2 Department of Ophthalmology, Xiangya Hospital, Central South University, 410008 Changsha, Hunan China; 3 Department of Biomedical Engineering, Georgia Institute of Technology/Emory University, Atlanta, GA 30332 USA; 4 Department of Cell Biology, University of Pittsburgh, Pittsburgh, PA 15213 USA; 5 McGowan Institute for Regenerative Medicine, University of Pittsburgh, Pittsburgh, PA 15213 USA; 6 Department of Developmental Biology, University of Pittsburgh, Pittsburgh, PA 15213 USA

**Keywords:** Mesenchymal stem cells, Glaucoma

Correction to: *Communications Biology* 10.1038/s42003-018-0227-z, published online 6 December 2018.

The original version of this Article omitted from the author list the 4^th^ author, Ajay Kumar, who is from ‘Department of Ophthalmology, University of Pittsburgh, Pittsburgh, PA 15213, USA’. Consequently, the Author Contributions were updated to the following, with the new text indicated in bold:

H.Y., Y.W., Y.Z., and Y.D. designed research; H.Y., Y.W., Y.Z., **A.K**., K.W., M.S., and Y.D. performed research; H.Y., Y.W., Y.Z., **A.K**., K.W., M.S., D.B.S., X.X., C.R.E., and Y.D. analyzed the data; and H.Y., Y.W., Y.Z., **A.K**., C.R.E., and Y.D. wrote the paper.

The errors have been corrected in both the PDF and HTML versions of the Article.

