# Supplementary material for: Human stem cells home to and repair laser-damaged trabecular meshwork in a mouse model
Source: Commun Biol. 2018 Dec 6;1:216. doi: 10.1038/s42003-018-0227-z (PMC6283842; doi:10.1038/s42003-018-0227-z)
Supplement: Supplementary file 1 — Description of Additional Supplementary Files [file 42003_2018_227_MOESM1_ESM.docx]

**Description of Additional Supplementary Files**

**File Name**: Supplementary Data 1

**Description**: An excel file of the original data of Figure 4.

**File Name**: Supplementary Data 2

**Description**: An excel file of the original data of Figure 7.
