# Supplementary material for: Human stem cells home to and repair laser-damaged trabecular meshwork in a mouse model
Source: Commun Biol. 2018 Dec 6;1:216. doi: 10.1038/s42003-018-0227-z (PMC6283842; doi:10.1038/s42003-018-0227-z)
Supplement: Supplementary file 2 — Supplementary Information [file 42003_2018_227_MOESM2_ESM.pdf]

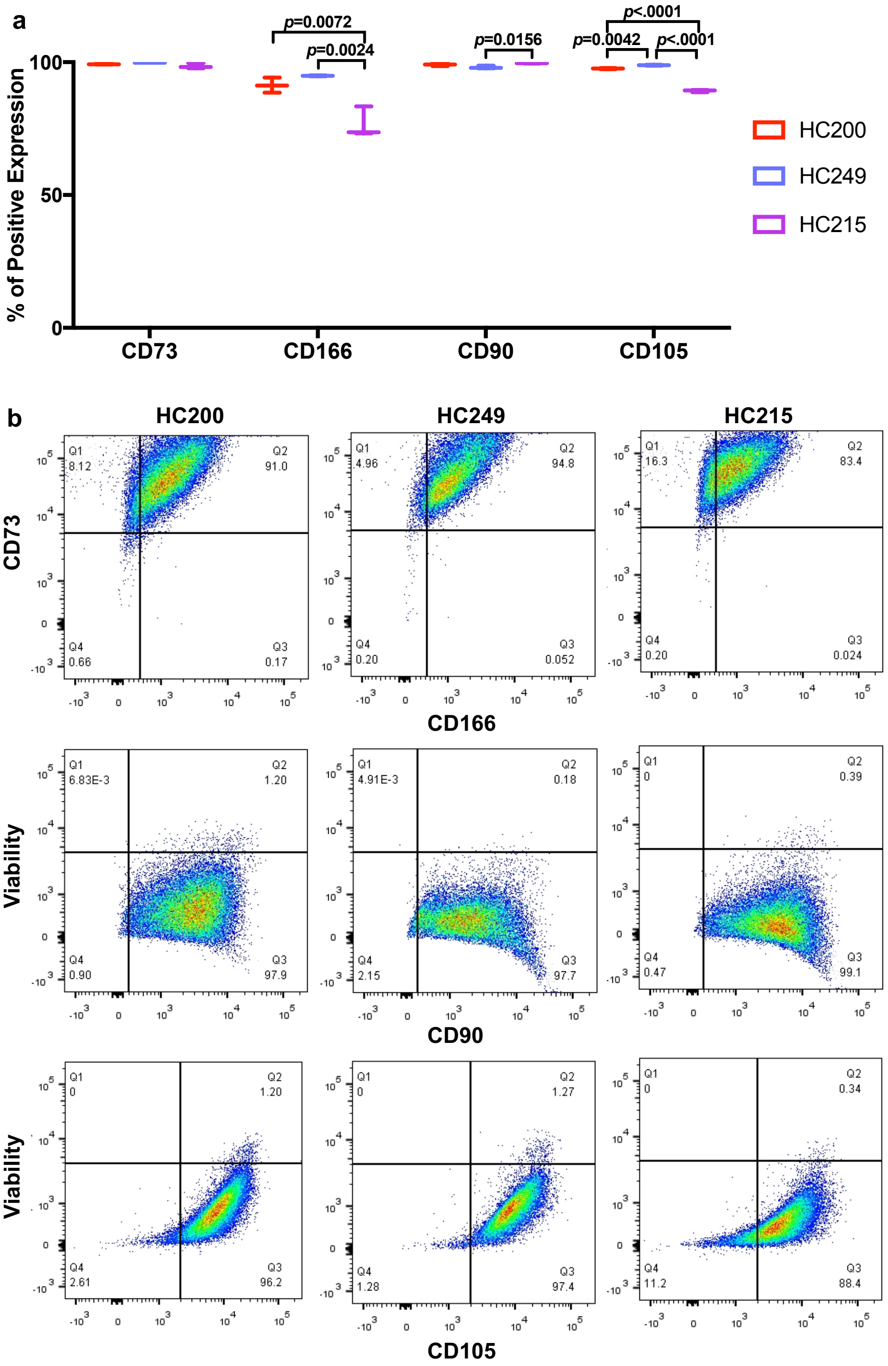

Fig. S1. Cell surface marker expression on human TMSCs. Human TMSCs used for in vitro and in vivo experiments were assessed for cell surface marker expression by flow cytometry. (**a**) Bars show the percentage of positive expression to CD73, CD166, CD90, CD105 on different cells from different donors (HC200, HC249, HC215). Statistical analysis was done using two-way ANOVA followed by a Tukey post test using SAS software. (**b**) Representative flow cytometry figures show Cluster of Differentiation marker expression and viability, as assessed by Live/Dead staining.

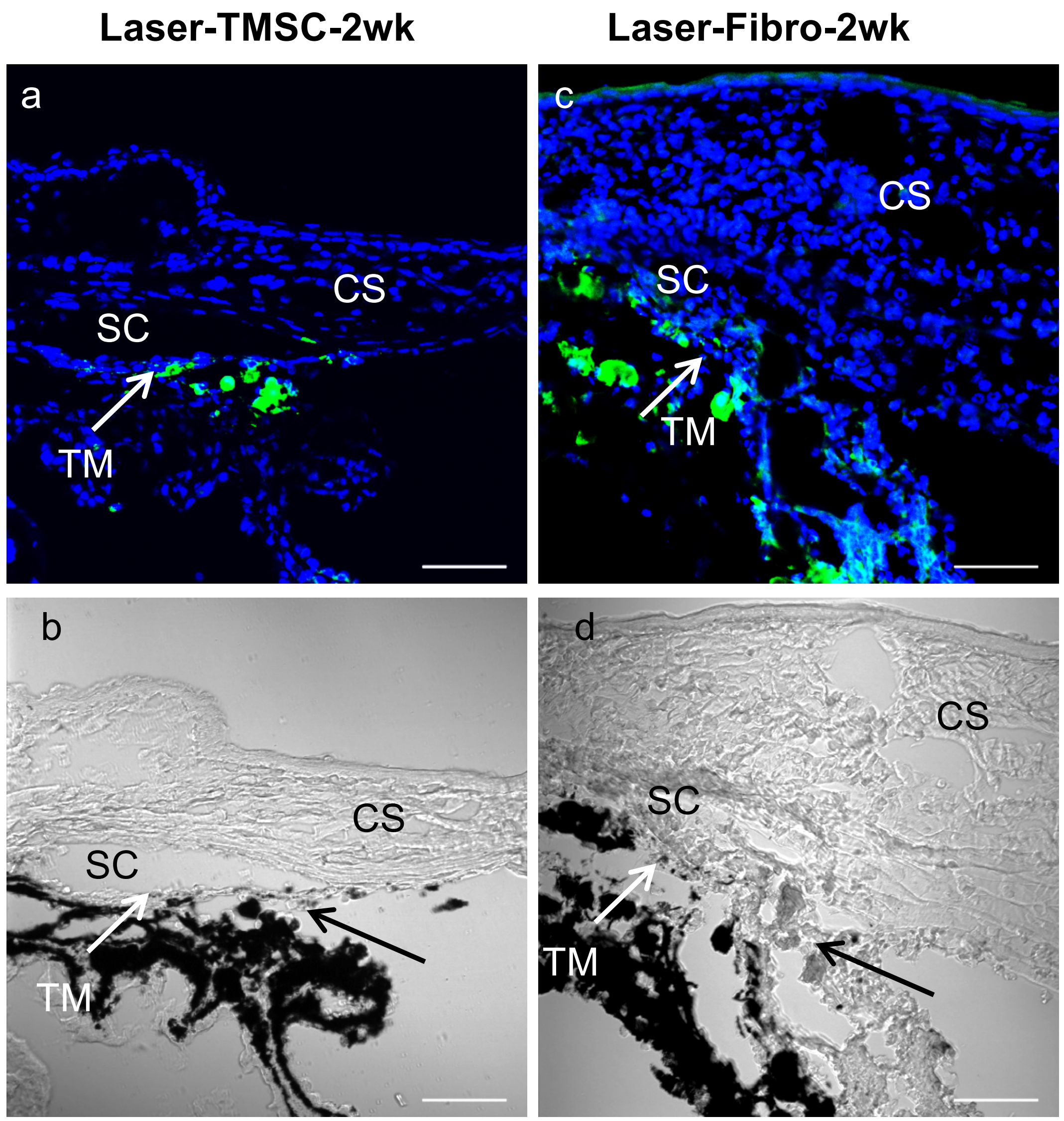

Fig. S2. Changes of anterior chamber angle after laser photocoagulation followed by intracameral cell transplantation. Cryosections show localization of DiO-labeled green TMSCs (**a**) or fibroblasts (**c**). DAPI stains nuclei blue. Bright field images with TMSC (**b**) or fibroblast transplantation (**d**) show the anterior chamber angle indicated by black arrows. White arrows point to the TM tissue where TMSC homed to (**a, b**) and where normal morphology was lost in fibroblast-transplanted eyes (**c, d**). Note that modest synechia are normal in C57BL/6 mouse eyes, but they were greatly enlarged in fibroblast-treated eyes. Scale bars, 50μm.

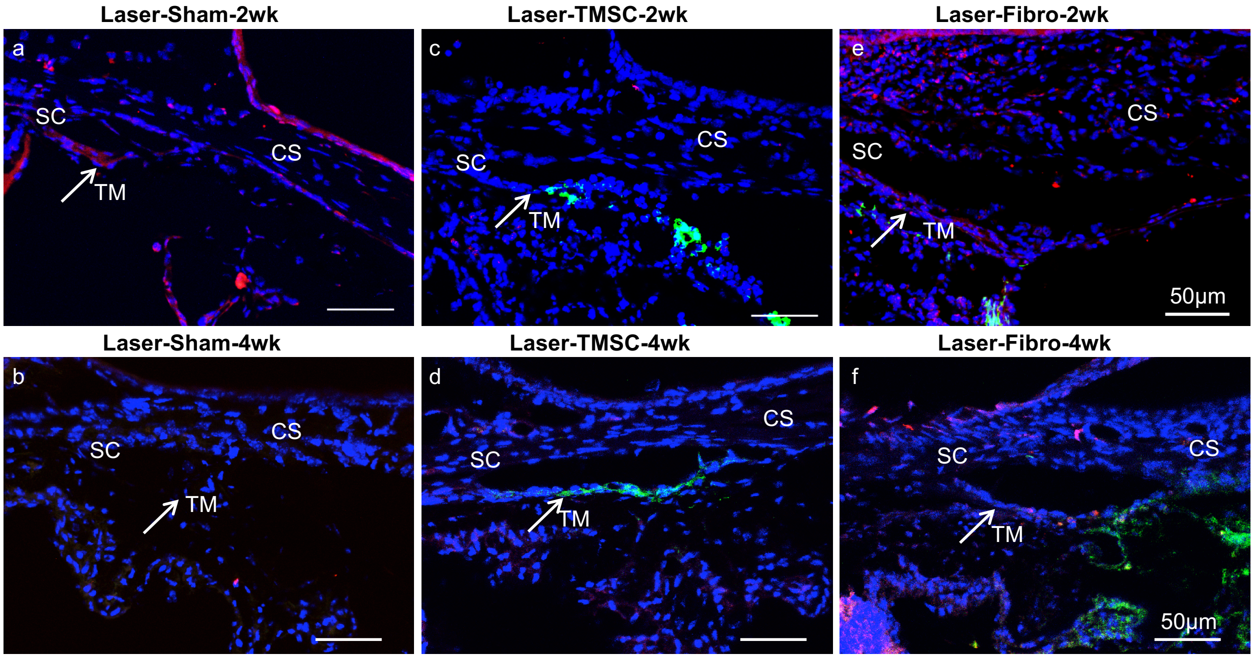

Fig. S3. Apoptotic cells in the TM after laser treatment. TUNEL staining was carried out on cryosections of eyes with laser + sham injection (**a, d**), laser + TMSC injection (**b, e**) and laser + fibroblast injection (**c, f**). **a, b** and **c** are at 2-wk after laser and injection. **d, e** and **f** are at 4-wk. Apoptotic cells were stained red. Injected TMSCs or fibroblasts were prelabeled with DiO (green). DAPI stains nuclei blue. Arrow points to the TM tissue. Scale bars, 50μm.

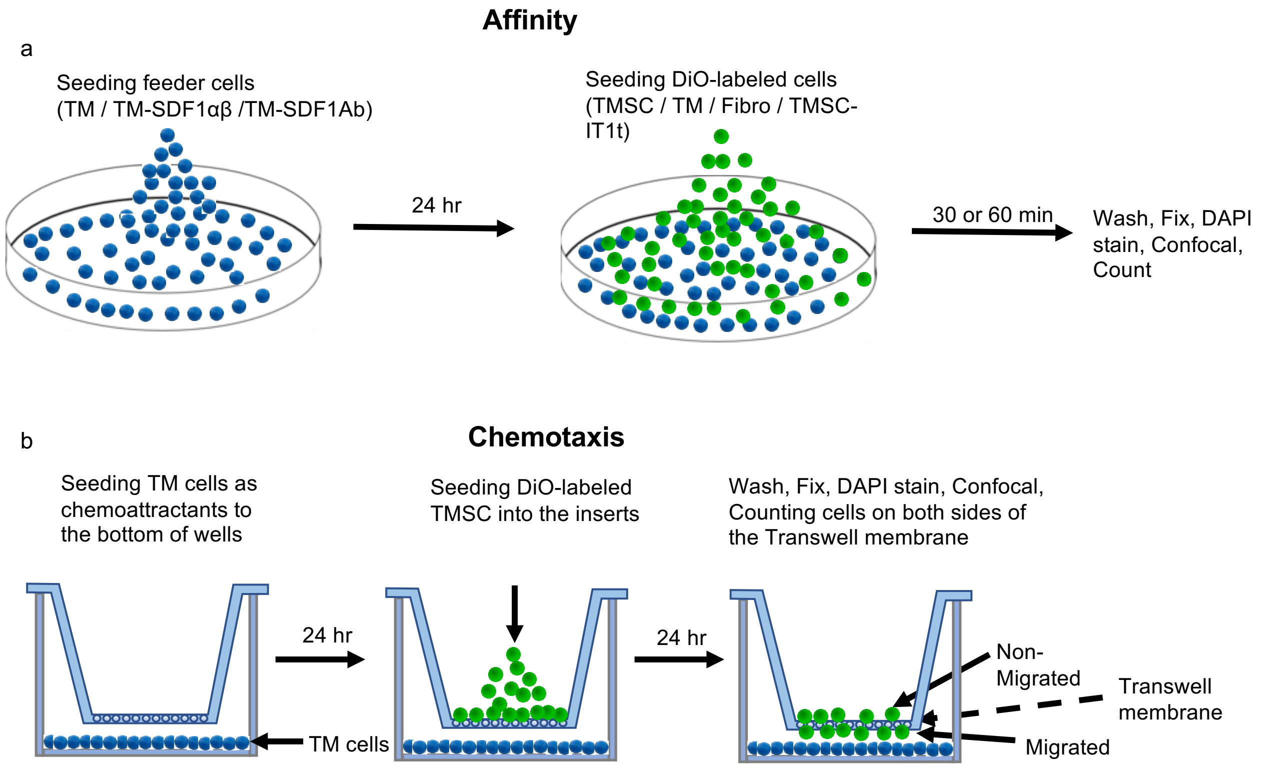

Fig. S4. Schematic illustration of affinity (a) and chemotaxis (b) assays.

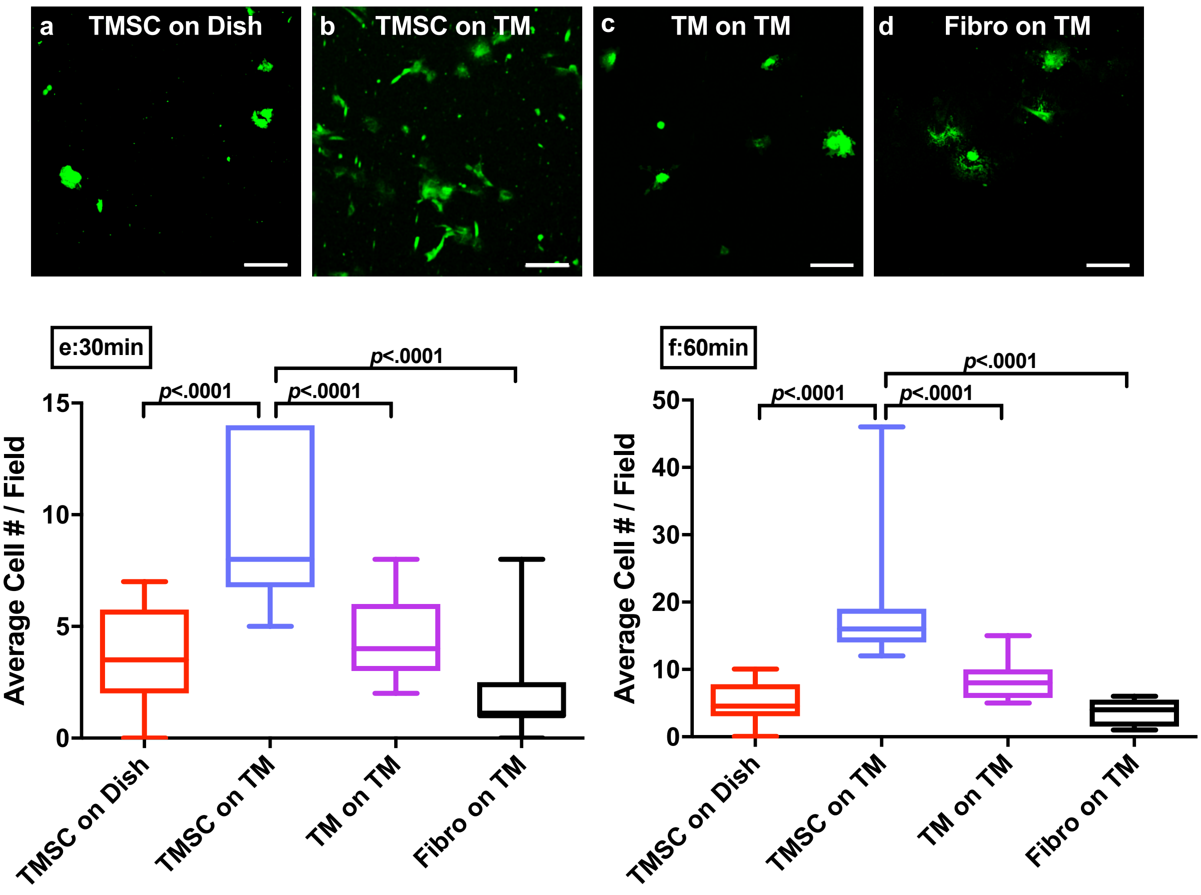

Fig. S5. Cell affinity depends strongly on cell pairing. (**a-d**) Representative figures showing cell attachment at 60 min. DiO-labeled green TMSCs were seeded in culture dishes without feeders (**a**) or on pre-seeded TM cells as feeders (**b**). (**c**) DiO-labeled green TM cells were seeded onto pre-seeded TM cells without labeling as feeders. (**d**) Fibroblasts were seeded on TM cells without labeling as feeders. Attached cells were counted and averaged from 5 fields of view at 30 min (**e**) and 60 min (**f**) after seeding. Scale bars, 100μm.

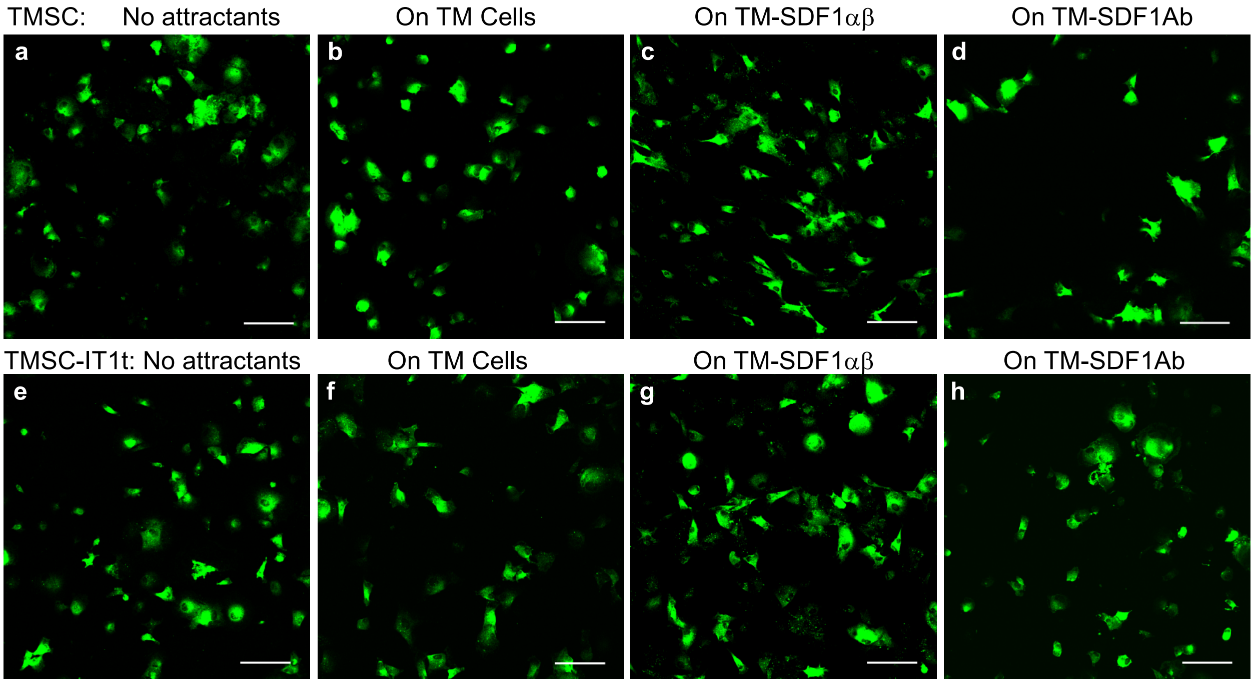

Fig. S6. TMSC affinity for TM cells with different SDF1 expression levels. Representative figures of DiO-labeled untreated TMSCs (**a-d**) or DiO-labeled TMSCs with reduced CXCR4 expression (TMSC-IT1t, treated with CXCR4 antagonist IT1t) (**e-h**), 60 min after seeding. The feeder conditions were cells seeded: directly on the dish without feeders (**a, e**), seeded on TM cells (**b, f**), seeded on TM-SDF1αβ cells (**c, g**; TM cells treated with SDF1α + 1β to increase SDF1 expression), or seeded on TM-SDF1Ab cells (**d, h**; TM cells treated with SDF1 antibody to reduce SDF1 expression) as feeders. The average cell counts and statistical analysis are shown in Fig. **7c** and **7d**. Scale bars, 100μm.

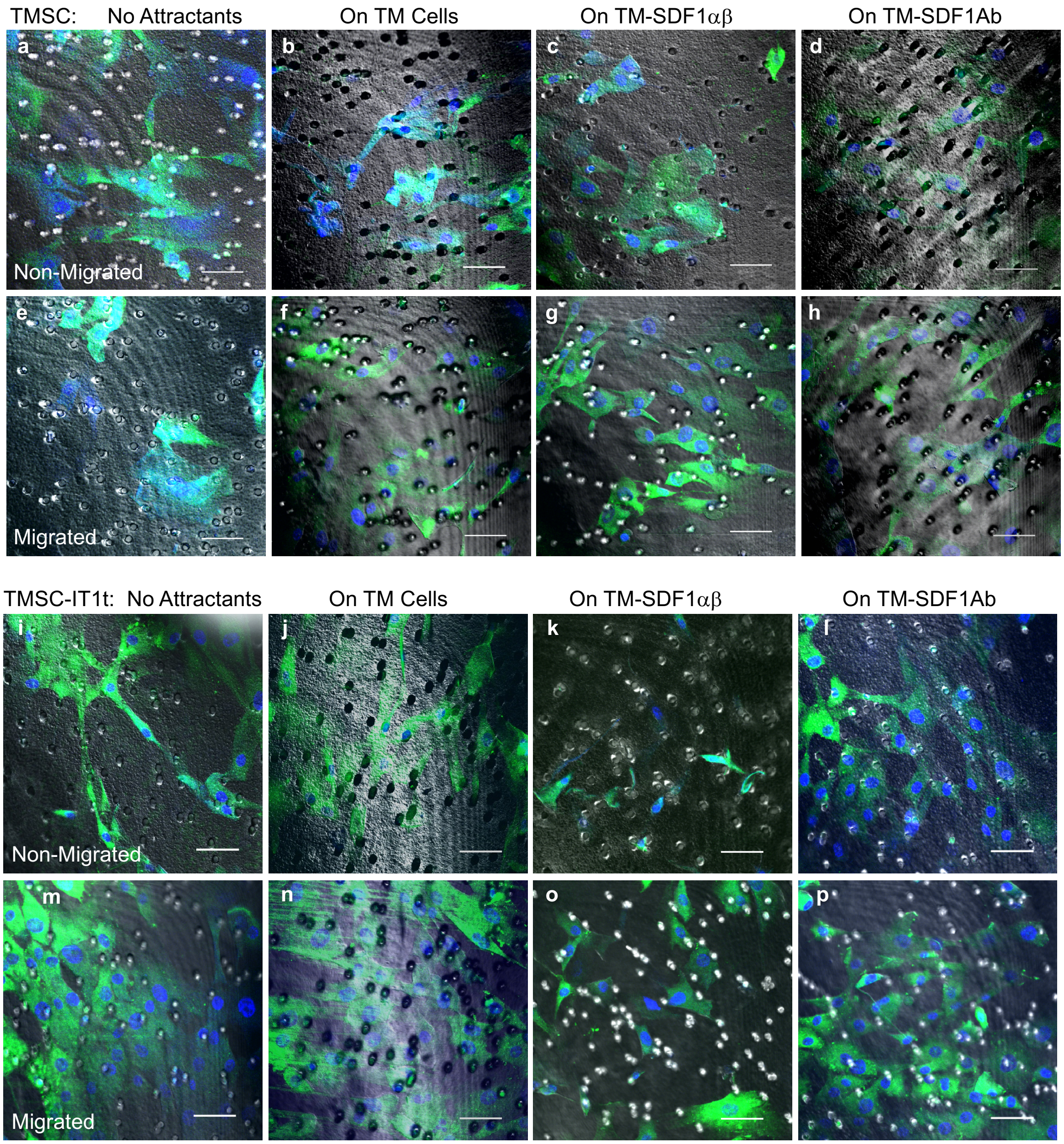

Fig. S7. Representative images of chemotaxis. DiO-labeled TMSCs (green, **a-h**) or DiO-labeled TMSCs with reduced CXCR4 expression (TMSC-IT1t, **i-p**) were seeded on top of Transwell membranes with different attractants on the bottom of cell culture plates. The attractants were as follows: no attractants (**a, e, i, m**), TM cells (**b, f, j, n**), TM-SDF1αβ cells (**c, g, k, o**) or TM-SDF1Ab cells (**d, h, l, p**). Images **a-d** and **i-l** show cells remaining on top of the transwell membranes without migration (“Non-Migrated”). Images of **e-h** and **m-p** show cells that migrated through the Transwell membrane pores (“Migrated”). DAPI stains nuclei blue. The black and white dots are the 8-μm pores of the Transwell membrane which allow cells to migrate. Statistical analysis is shown in Fig. 7e and 7f. Scale bars, 50μm.

Table S1. Primary antibodies used for immunostaining and Western blotting.

	Antibody	Type/color	Source	Catalog#
Primary Antibody	CHI3L1	Goat polyclonal	R&D	AF2599
	MGP	Mouse monoclonal	Santa Cruz	Sc-81546
	AQP1	Rabbit polyclonal	Santa Cruz	Sc-20810
	Myocilin	Rabbit polyclonal	Santa Cruz	Sc-20976
	OCT4	Rabbit polyclonal	Santa Cruz	Sc-9081
	SPARC	Goat polyclonal	R&D Systems	AF942
	CD45	Rat monoclonal	BD Biosciences	553076
Secondary Antibody	goat-anti-mouse	Alexa-555	ThermoFisher	A28180
	goat-anti-rabbit	Alexa-555	ThermoFisher	A27039
	goat-anti-rabbit	Alexa-647	ThermoFisher	A27040
	donkey-anti-rabbit	Alexa-546	ThermoFisher	A10040
	donkey-anti-goat	Alexa-633	ThermoFisher	A21082
	donkey-anti-rat	Alexa-555	Abcam	Ab150154

Table S2. Primer sequences used in quantitative RT-PCR

Gene Name	DNA Sequence
18S Ribosomal RNA	Forward: CCCTGTAATTGGAATGAGTCCAC<br>Reverse: GCTGGAATTACCGCGGCT
Human OCT4	Forward: GTGGAGGAAGCTGACAACAA<br>Reverse: GGTTCTCGATACTGGTTCGC
Human CHI3L1	Forward: GATGTGACGCTCTACGGCAT<br>Reverse: TGATGAAAGTCCGGCGACTC
Human Myocilin	Forward: AAGCCCACCTACCCCTACAC<br>Reverse: TCCAGTGGCCTAGGCAGTAT
Human CXCR4	Forward: CCTCAAGACCACAGTCATCC<br>Reverse: CTCAAACTCACACCCTTGCT
Human SDF1	Forward: TGTATCAGGACCCAGAGGAA<br>Reverse: ACACACCACAGCACAAACAC
Mouse CD45	Forward: ATGGTCCTCTGAATAAAGCCCA<br>Reverse: TCAGCACTATTGGTAGGCTCC
Mouse CD11b	Forward: ATGGACGCTGATGGCAATACC<br>Reverse: TCCCCATTCACGTCTCCCA
Mouse F4/80	Forward: CTTTGGCTATGGGCTTCCAGTC<br>Reverse: GCAAGGAGGACAGAGTTTATCGTG
Mouse SPARC	Forward: AAGATGTCCCTCAGCCTGG<br>Reverse: CCAGTGGACAGGGAAGATGT
Mouse FN	Forward: AGCAAGGAAAGTCACCCAGA<br>Reverse: TGTTTGACACACAGCCACAG
Mouse CXCR4	Forward: TCCTGCCCACCATCTACTTC<br>Reverse: CCGTCATGCTCCTTAGCTTC
Mouse SDF1	Forward: GCTCTGCATCAGTGACGGTA<br>Reverse: AGATGCTTGACGTTGGCTCT
Mouse Col3α1	Forward: ATCTGCAGAGCAACGGTCAT<br>Reverse: TGTTCTGACCAGTTGAGGTAGT
Mouse Col4α6	Forward: GGCTTGGGACTGTGATGCAA<br>Reverse: TCCCACATGGCTTTCCATAGG

Mouse αSMA	Forward: CCCTGAAGAGCATCCGACAC<br>Reverse: AGAGGCATAGAGGGACAGCA
Mouse TGFβ2	Forward: AGTTCAGGGTCTTCCGCTTG<br>Reverse: TGCTATCGATGTAGCGCTGG
